# Novel extracellular vesicle release pathway facilitated by toxic superoxide dismutase 1 oligomers

**DOI:** 10.1016/j.nbd.2026.107309

**Published:** 2026-02-04

**Authors:** Brianna Hnath, Srinivasan Ekambaram, Nikolay V. Dokholyan

**Affiliations:** a Department of Pharmacology, Penn State College of Medicine, Hershey, PA, USA; b Department of Biomedical Engineering, Pennsylvania State University, University Park, PA, USA; c Department of Neurology, The University of Virginia, School of Medicine, Charlottesville, VA, USA; d Department of Neuroscience, The University of Virginia, School of Medicine, Charlottesville, VA, USA; e Department of Biomedical Engineering, The University of Virginia, School of Medicine, Charlottesville, VA, USA

**Keywords:** ALS, Oligomer, Aggregation, SOD1, Extracellular vesicles, Spreading

## Abstract

Amyotrophic lateral sclerosis (ALS) is a fatal neurodegenerative disease that results in paralysis and death within three to five years. Mutations in over forty different proteins have been linked to ALS, raising debate over whether ALS is a single disease or multiple disorders with similar symptoms. Mutations in Cu,Zn superoxide dismutase 1 (SOD1) are found in only 2–3% of ALS cases, yet misfolded SOD1 appears in both sporadic (sALS) and familial (fALS) patients. Furthermore, mutations in TDP-43 or FUS increase levels of misfolded SOD1 on extracellular vesicles (EVs). Small EVs isolated from ALS patient samples have been shown to cause death of wild-type motor neurons and myotubes, supporting the theory that EVs play a role in spreading disease. We hypothesize that the previously identified toxic trimeric SOD1 spreads via EVs in ALS and influences the distribution of other ALS-related proteins, suggesting a common mechanism. To test this, we isolate EVs from motor neuron-like cells expressing mutations that stabilize trimers. We then perform a sandwich enzyme-linked immunosorbent assay (ELISA) using a CD9 capture antibody to measure whether misfolded SOD1 and 17 other ALS-related proteins increase or decrease on EVs with trimer stabilization. We identify which EV release pathway is affected by trimeric SOD1 using endocytosis and exocytosis inhibitors and analyze altered protein interaction pathways through co-immunoprecipitation and mass spectrometry proteomics. Our results show that VAPB, VCP, and Stathmin-2 increase on EVs when trimers are stabilized. The common pathway linking these ALS-associated proteins and SOD1 appears to involve multiple mechanisms, including the Caveolae endocytosis pathway, pointing to a novel hybrid EV release pathway in ALS. Overall, our findings show that trimeric SOD1 influences EV cargo and spread in ALS.

## 1. Introduction

Over 5000 people are diagnosed with ALS each year in the United States each year. Only 10% of these cases are familial, while the remaining 90% are sporadic cases (Redler and Dokholyan, 2012; Choi and Dokholyan, 2021). Similar proteins are found to be misfolded in both fALS and sALS. Since 1993, mutations in more than forty different genes have been linked to ALS, and more continue to be identified (Vildan et al., 2019). The majority of the genetic mutations associated with ALS fall into four main functional categories: RNA processing, protein trafficking, cytoskeletal/axonal dynamics, and mitochondrial function (Nguyen et al., 2021). SOD1 is distinctive in that misfolded SOD1 has been shown to impact processes across all four categories. Many ALS-related proteins form large, insoluble aggregates when misfolded. Initially, large aggregates were thought to transmit extracellularly, spreading ALS between cells (Arnold et al., 2023). Many studies focus on the potential prion-like misfolded propagation of ALS-related proteins without proposing a common mechanism of spread (McAlary et al., 2019; Arnold et al., 2023). Recently, smaller soluble forms of disease-associated proteins have been identified as toxic, while the larger insoluble aggregates are considered protective to cells (Hnath et al., 2023). As these soluble misfolded proteins are detected in or on EVs, we posit that EVs may play a crucial role in the spread of ALS, and this theory is becoming more prevalent Afonso et al., 2023).

SOD1 is a cytosolic antioxidant enzyme that naturally forms a dimer and converts superoxide radicals into oxygen and hydrogen peroxide (Nguyen et al., 2021). Mutations in SOD1 destabilize the dimer interface, leading to the loss of metals and aggregation into both soluble and insoluble oligomers (Khare et al., 2004). Mutant SOD1 (G93A) mouse models exhibit a loss of motor function, while SOD1 knockdown mouse models do not demonstrate the same phenotype (Gurney et al., 1994; Reaume et al., 1996). This study led to the conclusion that SOD1 misfolding results in a toxic gain-of-function rather than a loss of function. While mutations in SOD1 are found in only 1–3% of all patients, misfolded SOD1 has been observed among patients with other ALS-related mutations (such as fused in sarcoma (FUS) and TDP-43) as well as in sporadic patients (Zeineddine et al., 2015; Jeon et al., 2019; Paré et al., 2018; Forsberg et al., 2010). Several other factors beyond mutations cause SOD1 to destabilize and aggregate, including post-translational modifications (primarily glutathionylation)(Redler et al., 2011; Banks and Andersen, 2019; Wilcox et al., 2009), loss of metals (Khare et al., 2004; Khare and Dokholyan, 2006), crowding from overexpression (Nguyen et al., 2021), and environmental toxins (such as BMAA and ammonia)(Khare et al., 2004; Xu et al., 2018; Sutedja et al., 2009; Proctor et al., 2019; Hnath and Dokholyan, 2022). The presence of misfolded SOD1 in sporadic and non-SOD1 familial ALS patients has been highly debated over the past decade, likely due to inconsistencies in the antibodies used (Paré et al., 2018; Atlasi et al., 2018; Rotunno and Bosco, 2013) and variations in tissue types and preparations. Forsberg, Pare, Grad, and Pokrishevsky all observed misfolded SOD1 in non-SOD1 fALS and sALS patient spinal cord sections using either immunohistochemistry or immunoprecipitation (Paré et al., 2018; Forsberg et al., 2010; Pokrishevsky et al., 2016; Grad et al., 2011). The difficulty of antibodies in identifying smaller oligomers of SOD1 has sustained the failed theory that large insoluble aggregates are responsible for the progression of ALS (Hnath and Dokholyan, 2022; Zhu et al., 2018).

When the SOD1 dimer interface is disrupted, SOD1 aggregates and forms a wide range of soluble oligomers and insoluble fibrils (Wilcox et al., 2009; Redler et al., 2014; Khare et al., 2004; Khare and Dokholyan, 2006). Most previous studies consider misfolded SOD1 to mean a mix of all aggregate sizes, yet the different-size oligomers have drastically different toxicities and structures. In 2016, Proctor et al. determined that small soluble trimeric oligomers of SOD1 had the highest toxicity in cell models and that the toxicity correlated with the amount of thermodynamic stability of trimeric SOD1(Proctor et al., 2016). Zhu et al. further demonstrated that larger SOD1 oligomers and insoluble fibrils are protective to cells (Zhu et al., 2018). In 2022, Hnath and Dokholyan studied the aggregation mechanism of SOD1 using structural characterization assays and determined that trimeric SOD1 is a structurally independent species that forms in direct competition with larger aggregates, as opposed to trimeric SOD1 being a preliminary step in larger aggregate formation (Hnath and Dokholyan, 2022). Due to aggregate structural differences, very few anti-misfolded SOD1 antibodies bind to trimeric SOD1; C4F6 is the only antibody we have identified that recognizes trimeric SOD1(Atlasi et al., 2018; Redler et al., 2014; Proctor et al., 2016). The inability of most antibodies to detect trimeric SOD1 may account for the drastic inconsistencies between studies quantifying misfolded SOD1 in patient samples.

The mechanism of the spread of ALS throughout the body is largely unknown. EV release is increasingly recognized as a key mechanism in ALS pathogenesis, particularly in the spread of pathological proteins and disease-associated toxicity between cells (Afonso et al., 2023; Anakor et al., 2021). EV biogenesis involves intricate endocytic and exocytic pathways that regulate cargo uptake, processing, and secretion, and dysfunction in these pathways may underlie the propagation of misfolded proteins such as SOD1 in ALS. Normally, intracellular proteins destined for EVs originate from the ER-Golgi network or are internalized through caveolin-dependent endocytosis, clathrin-dependent endocytosis, or pinocytosis. The proteins then traffic through early and late endosomes before being sorted for either lysosomal degradation or secretion (Abels and Breakefield, 2016). However, in ALS, impaired endolysosomal processing may shift this balance, leading to excessive secretion of neurotoxic proteins on EVs rather than degradation (Afonso et al., 2023).

Misfolded SOD1 has been identified on the surface of EVs secreted from different ALS cell models (HEK293, Neuro2a, or NSC-34)(Grad et al., 2014; Urushitani et al., 2006; Gomes et al., 2007) or from ALS mouse models (Urushitani et al., 2006; Silverman et al., 2019). Misfolded SOD1 was found colocalized with neurosecretory vesicle proteins chromogranin A and B, indicating that misfolded SOD1 is secreted by cells instead of being released solely through apoptosis (Urushitani et al., 2006). Small EVs from ALS patients were observed to be neurotoxic when added to stem cell-derived motor neuron and muscle cell cultures, yet larger EVs had no effect on viability (Anakor et al., 2022), suggesting the deadly progression of ALS may be vesicle size dependent. Overexpression of SOD1 mutants may impair ER-Golgi transport, leading to Golgi fragmentation and ER stress (Bellouze et al., 2016; Atkin et al., 2014). Rab guanosine triphosphatases (GTPases) regulate all membrane trafficking events, and Rab5, Rab7, and Rab11 have been implicated in ALS (Parakh et al., 2018). Rab7 and Rab11 co-localize with the ALS-associated protein C9orf72(Farg et al., 2014), which is expected since Rab7 controls *endo*-lysosomal transport and Rab11 regulates recycling endosomes—both essential for clearing misfolded proteins (Kiral et al., 2018). Mutations in ALS-associated Alsin (ALS2) disrupt Rab5 activation, impairing endosomal trafficking and vesicle biogenesis, which Rab5 normally regulates by facilitating early endosome fusion and cargo sorting (Hadano et al., 2010). Consequently, enlarged Rab5-positive endosomes are observed in spinal ALS motor neurons (Lai et al., 2009). Understanding these dysregulated pathways could uncover new therapeutic targets aimed at disrupting the intercellular spread of ALS pathology. Beyond ALS2, multiple mutated genes found in ALS patients are involved in vesicular trafficking and have been shown to affect or coincide with SOD1 misfolding (McCluskey et al., 2022). These genes can be grouped into four main functional categories as mentioned previously (Nguyen et al., 2021): RNA processing—fused in sarcoma (FUS)(Pokrishevsky et al., 2016) and TAR DNA-binding protein 43 (TDP-43)(Pokrishevsky et al., 2016); protein trafficking–C9orf72(Forsberg et al., 2010), vesicle-associated membrane protein-associated protein B (VAPB)(Forsberg et al., 2010; Teuling et al., 2007), Sequestosome-1/p62 (SQSTM1)(Mitsui et al., 2022; Hadano et al., 2016), Ubiquilin 2 (UBQLN2)(Wu et al., 2020), Valosin-containing protein (VCP)(Tsioras et al., 2023), Optineurin (OPTN), and TANK-binding kinase 1 (TBK1)(Gerbino et al., 2020); cytoskeletal/axonal dynamics – ALS2(Forsberg et al., 2010; Hadano et al., 2016; Li et al., 2011), charged multivesicular body protein 2B (CHMP2B), Spatacsin (SPG11), Stathmin-2(Bellouze et al., 2016), and NIMA-related kinase 1 (NEK1); and mitochondrial function – polyphosphoinositide phosphatase (Fig. 4), NSFL1, and cyclin-dependent kinase 5 (Cdk5). We propose that misfolded, trimeric SOD1 is a central component of the mechanism of spreading of ALS on EVs, offering novel therapeutic targets to alter the disease progression.

To elucidate the pathways involved in the spread of toxic SOD1 and other ALS-associated proteins on EVs, we established a system to test the effect of SOD1 trimer on other proteins that do not solely rely on the use of antibodies (due to the difficulty for most antibodies to bind to trimeric SOD1). We used previously designed mutations that stabilize the trimeric form of SOD1 as well as the common disease mutation A4V which partially stabilizes trimeric SOD1 and determined that stabilizing trimeric SOD1 leads to an increase in misfolded SOD1 on EVs. We used this method to test whether the stabilization of the SOD1 trimer affects the levels of a panel of other ALS-associated proteins on EVs. Upon narrowing the panel down to three main proteins (VAPB, VCP, and Stathmin-2), we determined the effect of six different exocytosis and endocytosis inhibitors on the levels of VAPB, VCP, and Stathmin-2 with trimer stabilization. Using co-immunoprecipitation mass spectrometry proteomics, we further identified protein pathways that interact with VAPB, VCP, and Stathmin-2 and are altered by trimeric SOD1 stabilization. Additionally, we tested the effect of trimer stabilization on a panel of twenty-two different EV-related proteins. We determined a novel hybrid pathway responsible for the release of altered EVs with trimeric SOD1 stabilization, shedding light on molecular events driving the disease propagation.

## 2. Results

### 2.1. Trimeric SOD1 stabilization increases misfolded SOD1 on the surface of EVs

**Fig. 1.**
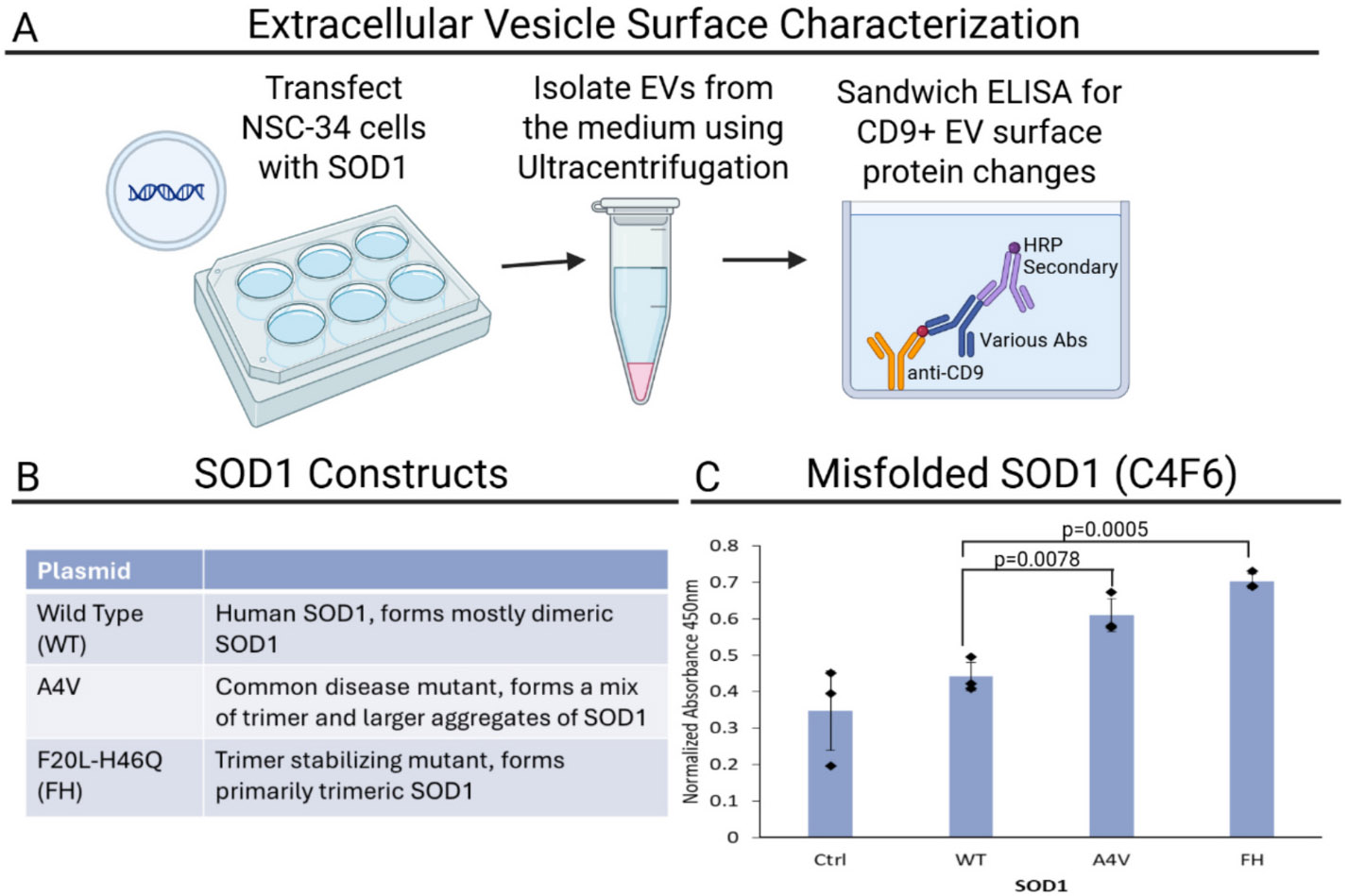
Stabilization of trimeric SOD1. A) Schematic diagram illustrating the workflow of transfecting NSC-34 cells with SOD1, isolating extracellular vesicles (EVs) from the culture medium via ultracentrifugation, and analyzing CD9+ EV surface protein changes using a sandwich ELISA. B) SOD1 plasmid mutations (or WT) to incrementally stabilize trimeric SOD1 in cells. C) Incrementally stabilizing trimeric SOD1 significantly increased the level of misfolded SOD1 on EVs isolated through an anti-CD9 sandwich ELISA. WT to A4V *p*-value = 0.0078, WT to FH p-value = 0.0005.

We hypothesize that cytotoxic trimeric SOD1 is spreading on EVs throughout ALS and altering the spread of other ALS-related proteins. Grad et al. have previously determined that misfolded SOD1 is present on the exterior of vesicles secreted by cells stably expressing WT or mutant SOD1(Grad et al., 2014; Grad et al., 2011) using atomic force microscopy with immunogold labeling for SOD1, but the conformation of misfolded SOD1 and other proteins being altered on EVs when SOD1 is misfolded has not been established. We exploited mutations that progressively increase trimer stabilization (A4V mildly stabilizes trimer formation, and F20L/H46Q (FH) strongly stabilizes trimer formation) (Hnath and Dokholyan, 2022) to determine if stabilizing trimeric SOD1 increases the concentration of misfolded SOD1 on EVs. The antibody C4F6 is the only antibody that binds trimeric SOD1(Proctor et al., 2016; Atlasi et al., 2018), but it also binds other misfolded SOD1 species, making it difficult to isolate molecular effects due solely to SOD1 trimers. Incrementally stabilizing trimeric SOD1 in motor neuron-like NSC-34 cells circumvents this difficulty. We previously designed the FH mutant SOD1 molecule that promotes the stabilization of SOD1 in a toxic trimeric state (Hnath and Dokholyan, 2022) (Fig. 1A, B). Overexpression of FH in NSC-34 cells leads to a significant increase of misfolded SOD1 (anti-C4F6 antibody) concentration on EVs, compared to overexpression of wild type (WT) or a commonly found SOD1 mutation (A4V) (Fig. 1C, 1-way ANOVA F-statistic: 13.06, *p*-value: 0.0019, pairwise comparisons WT to A4V p-value: 0.0078, WT to FH p-value: 0.0005). Due to the previously established tendency for the FH mutations to promote trimer formation (Hnath and Dokholyan, 2022), the misfolded species being identified by C4F6 are likely trimeric SOD1. We utilize this system to determine pathways responsible for the spread of toxic trimeric SOD1 EVs.

EV isolation was performed using an initial 1000 x*g* centrifugation to remove cell debris, followed by a 2-h centrifugation at 100,000 x*g* to concentrate EVs into a pellet. This method of isolation focuses on high recovery with low specificity, since we then utilized a sandwich ELISA for tetraspanin CD9 to isolate EVs. Gomes in 2007 and Grad in 2014 demonstrated that hSOD1 is fully present in an initial 100,000 x*g* centrifugation of enriched cell medium, they performed an additional sucrose density gradient purification then selected for CD9+ fractions to obtain a purified sample for more specific assays such as nanoparticle tracking analysis for size quantification and atomic force microscopy (Gomes et al., 2007; Grad et al., 2014). Since we do not require a purified sample (and the additional purification significantly decreases yield), we did not perform the sucrose density gradient for our assays.

### 2.2. ALS-associated proteins increase and decrease on EVs with trimeric SOD1 stabilization

**Fig. 2.**
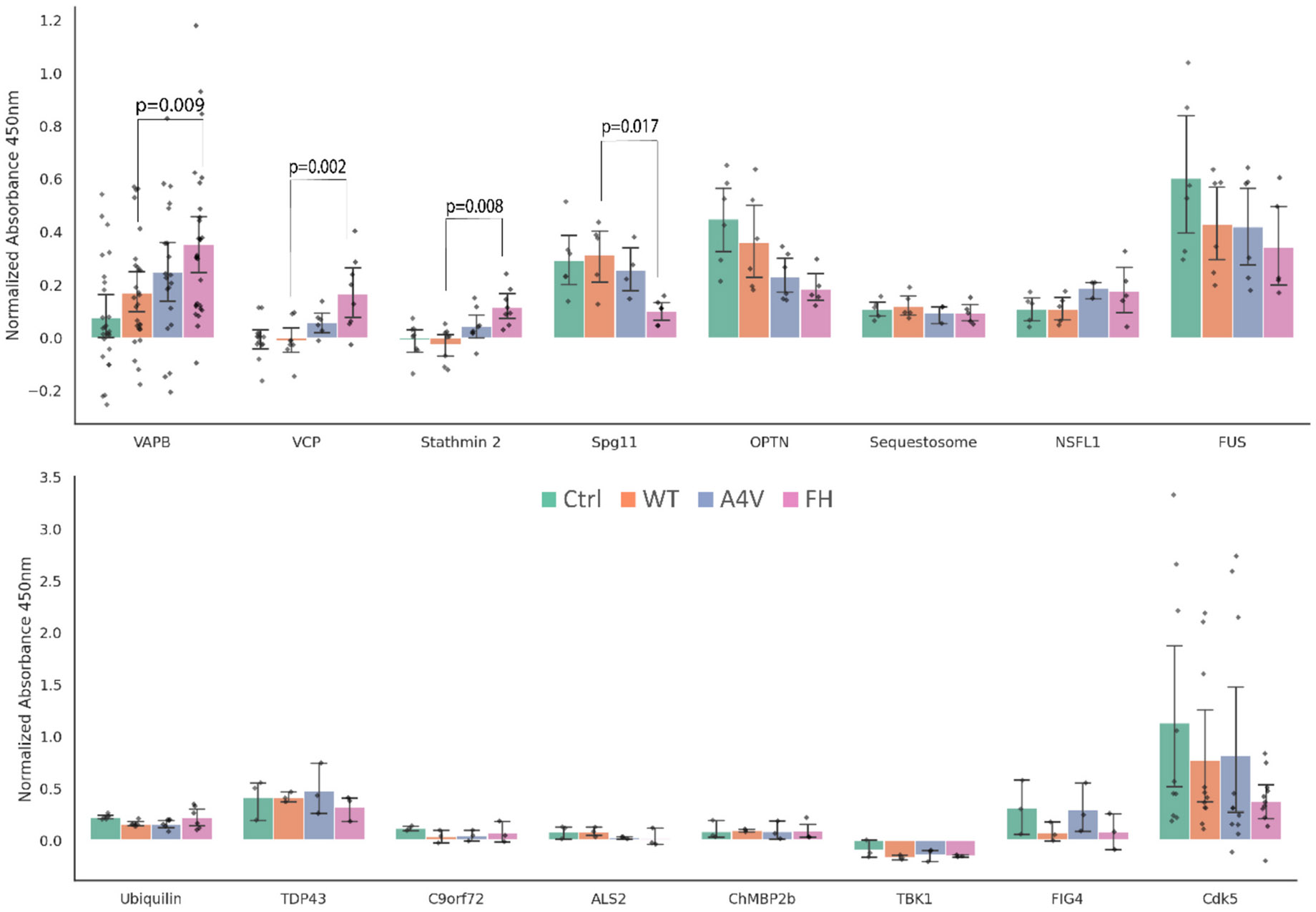
Effects of SOD1 trimer stabilization on ALS-associated proteins on CD9+ EVs. Expression levels of 16 ALS-associated proteins on CD9+ EVs were measured through a sandwich ELISA following incremental trimeric SOD1 stabilization (Control (Ctrl) cells had the lowest levels of trimeric SOD1 and FH (F20L-H46Q) mutant cells had the highest levels). Data are shown as bar graphs representing mean protein expression with overlaid points showing individual replicates. Stabilization of trimeric SOD1 resulted in significant increases in VAPB (p-value = 0.009), VCP (p-value = 0.002), and Stathmin-2 (p-value = 0.008). In contrast, Spg11 (p-value = 0.017) levels significantly decreased, while OPTN showed a non-significant trend towards decreased expression (p-value = 0.0546).

Many ALS-associated proteins are already known to be linked to EV formation and release (McCluskey et al., 2022). We selected a panel of 17 ALS-associated proteins (Fig. 2) that either had known associations with EVs or were the most commonly found ALS mutations (C9ORF72, TDP-43, and FUS) and used our incremental trimer stabilization system to determine if any ALS-associated proteins are altered on EVs due to trimeric SOD1. While many of the proteins tested displayed significant alterations using a one-way ANOVA test (VAPB: F = 6.19, *p* = 0.00068, VCP: F = 7.54, *p* = 0.00060, Stathmin 2: F = 7.53, *p* = 0.00076, Spg11: F = 4.88, *p* = 0.0126, OPTN: F = 4.14, *p* = 0.0204, NEK1: F = 10.75, *p* = 0.00353), only concentrations of VAPB, VCP, Stathmin-2, and SPG11 incrementally changed on EVs (with significant pairwise comparisons) from cells expressing trimeric SOD1(FH) compared to WT controls. Concentrations of VAPB (pairwise WT and FH p-value 0.009), VCP (pairwise WT and FH p-value 0.002), and Stathmin-2 (pairwise WT and FH p-value 0.008) increased, while concentrations of SPG11 (pairwise WT and FH p-value 0.017) and OPTN (not significant) decreased with trimer stabilization (Fig. 2). We did not observe significant incremental changes in the concentration of FUS, TDP-43, C9orf72, SQSTM1, UBQLN2, TBK1, ALS2, CHMP2B, NEK1, FIG. 4, NSFL1, and Cdk5 with trimer stabilization.

Since VAPB, VCP, and Stathmin-2 are all known to be involved in EVs (McCluskey et al., 2022; Thornburg-Suresh and Summers, 2024), we chose to focus on these three proteins for the remainder of the study while still taking into account all significant protein changes when considering potential EV release pathways. VAPB is a transmembrane protein involved in the tethering of the endoplasmic reticulum (ER) to other organelles, as well as the regulation of lipids (Kors et al., 2022; Borgese et al., 2021), VCP is broadly involved in vesicle trafficking and protein degradation (Scarian et al., 2022), and Stathmin-2 is the microtubule-associated protein responsible for destabilizing microtubules, allowing for axonal repair and maintenance, and has also been associated with Golgi fragmentation (Bellouze et al., 2016; Thornburg-Suresh and Summers, 2024). All three proteins are integral to different parts of EV formation and release, making it difficult to narrow down to a singular common pathway. We additionally tested whether ALS-associated mutations in VAPB and VCP caused the same increase in the proteins on EVs, but the mutants had no significant effects (Supplementary Fig. S2) (Stathmin-2 does not yet have any direct mutations linked to ALS, Stathmin-2 can undergo cryptic splicing by other ALS-associated proteins leading to a loss of function (Krus et al., 2022)). Next, we determine a common pathway of release for these three altered proteins.

### 2.3. Isolating the mechanism of release of altered EVs

To identify the pathway involved in the release of altered EVs due to trimer stabilization, we utilized three different methods. First, we used a panel of common endocytosis and exocytosis inhibitors (Catalano and O’Driscoll, 2020; Münch et al., 2011) to determine if inhibiting one pathway would affect the levels of VAPB, VCP, and Stathmin-2. Second, we performed co-immunoprecipitation of VAPB, VCP, and Stathmin-2 from cell lysate overexpressing either WT or trimeric SOD1, and utilized mass spectrometry proteomics to quantify any altered protein interactions. Third, we evaluated groups of proteins involved in vesicle docking/release, ER/Golgi transport, movement of vesicles by the cytoskeleton, and lysosomal sorting to further narrow down the common pathway involved (Liu and Wang, 2023).

**Fig. 3.**
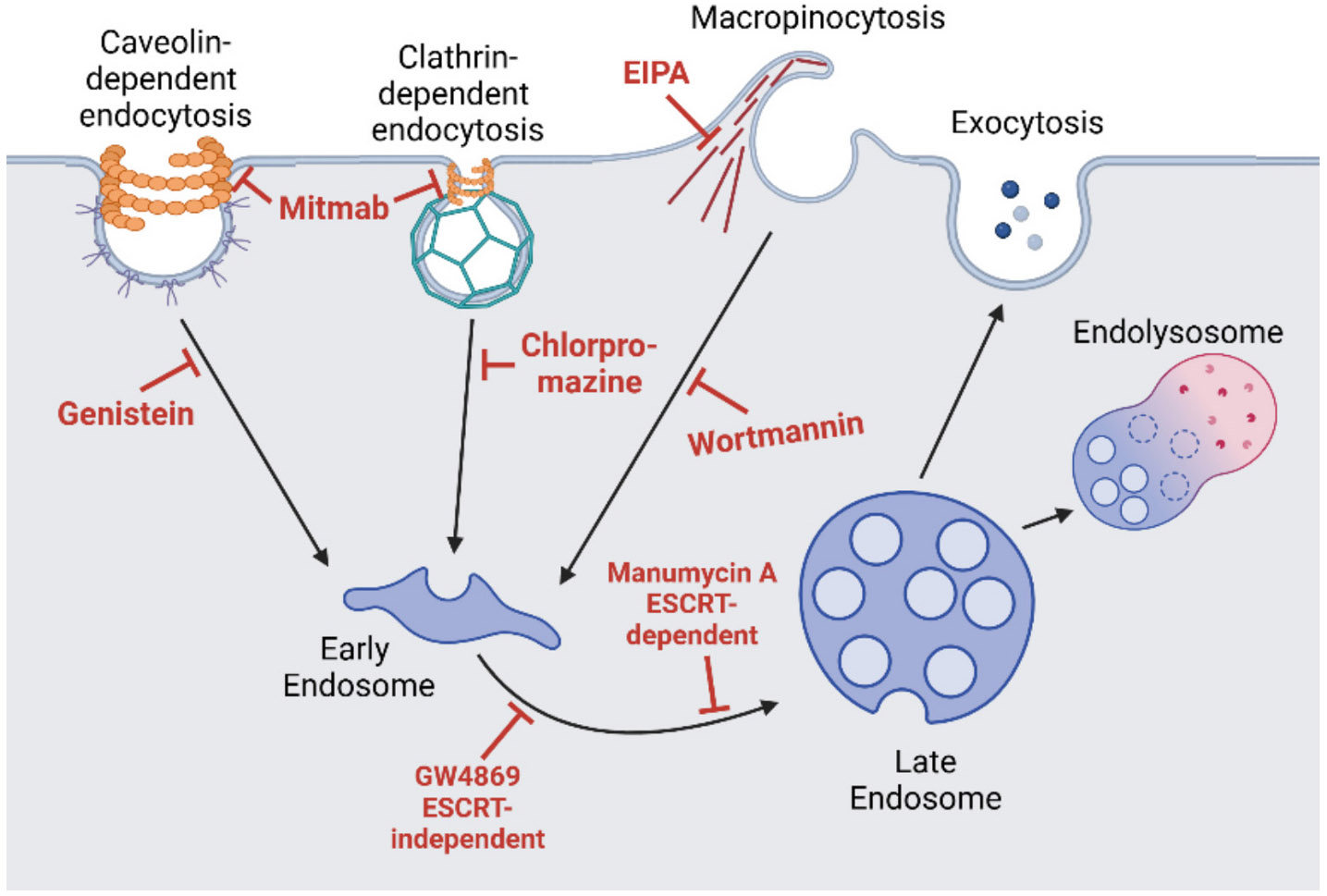
Six different endocytosis and exocytosis inhibitors were utilized to evaluate which EV pathways are being affected by trimeric SOD1 stabilization.

**Fig. 4.**
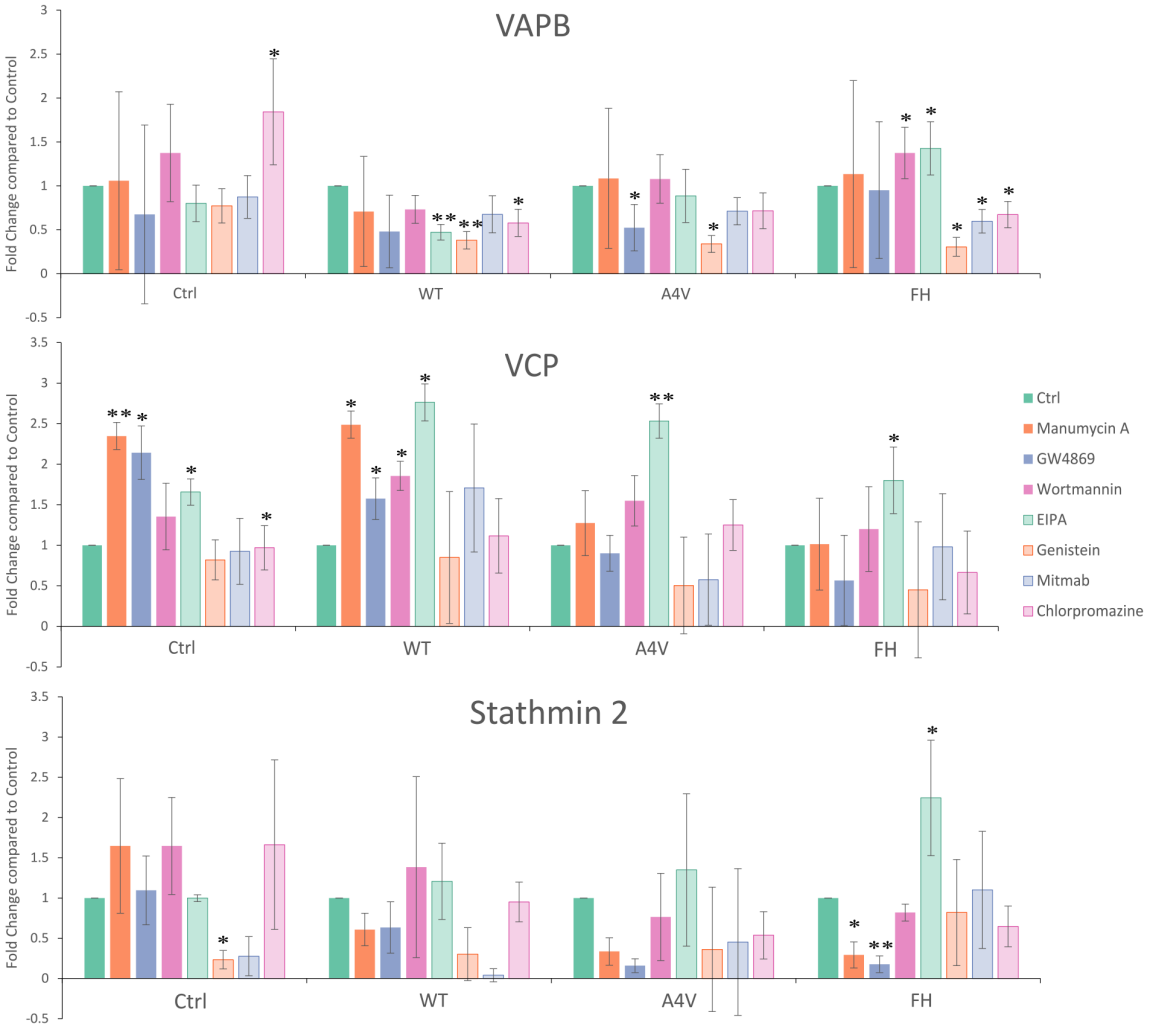
Exocytosis and endocytosis inhibitors differentially affect VAPB, VCP, and Stathmin-2 levels on CD9+ EVs following SOD1 trimer stabilization. NSC-34 cells with stabilized trimeric SOD1 were treated with inhibitors targeting ESCRT-dependent and independent exocytosis, macropinocytosis, and caveolin or clathrin-mediated endocytosis to identify pathways contributing to the release of trimer-altered EVs. Fold changes in VAPB, VCP, and Stathmin-2 levels on CD9+ EVs (determined through a sandwich ELISA) were calculated relative to a vehicle control (DMSO). Inhibition of macropinocytosis with EIPA resulted in a significant increase in all three proteins, whereas inhibition of caveolin-mediated endocytosis with Genistein reduced the protein levels on EVs. Other inhibitors showed protein-specific or non-significant effects. Bar graphs represent mean fold change ± absolute error (*n* = 3). * = p-value <0.05, ** = p-value <0.001.

Six different pathway inhibitors were tested; ESCRT-dependent exocytosis inhibitor Manumycin A, ESCRT-independent exocytosis inhibitor GW4869, macropinocytosis inhibitor Wortmannin, Na/H channel inhibitor (also a factor in macropinocytosis) ethyl isopropyl amiloride (EIPA), tyrosine kinase inhibitor (Caveolae-mediated endocytosis) Genistein, dynamin inhibitor MitMAB (affects both Caveolin-dependent and Clathrin-dependent endocytosis), and a clathrin endocytosis inhibitor Chlorpromazine were tested in NSC-34 cells (Fig. 3) (Catalano and O’Driscoll, 2020; Münch et al., 2011). If a certain pathway was involved in the release of the trimer-altered EVs, we expected to observe a decrease in VAPB, VCP, and Stathmin-2 when the release pathway is inhibited. Instead, the only inhibitor that caused a significant change in FH for all three proteins was EIPA, which caused a significant increase instead of a decrease (VAPB *p*-value = 0.0166, VCP p-value = 0.0503, and Stathmin-2 p-value = 0.0344). VAPB, VCP, and Stathmin-2 all followed a similar trend of having increased (or not significantly changed) concentrations on EVs with the addition of macropinocytosis inhibitors Wortmannin and EIPA (VAPB Wortmannin p-value = 0.0236, VCP and Stathmin-2 were non-significant), then having decreases on EVs (or not significantly changed) with the addition of caveolin and clathrin endocytosis inhibitors Genistein (VAPB p-value = 0.004), MitMab (VAPB p-value = 0.024), and Chlorpromazine (VAPB p-value = 0.025), as well as exocytosis inhibitors Manumycin A (Stathmin-2 p-value = 0.003) and GW4869 (Stathmin-2 p-value = 0.0007) (Fig. 4). The significant increase with inhibition of the macropinocytosis pathway could mean that an alternative endocytosis pathway is compensating for vesicular turnaround and leading to an increase in VAPB, VCP, and Stathmin-2 when trimer is stabilized. Either ESCRT-dependent or independent exocytosis pathways could be contributing to the trimer altered release as could the caveolin and clathrin endocytosis pathways.

**Fig. 5.**
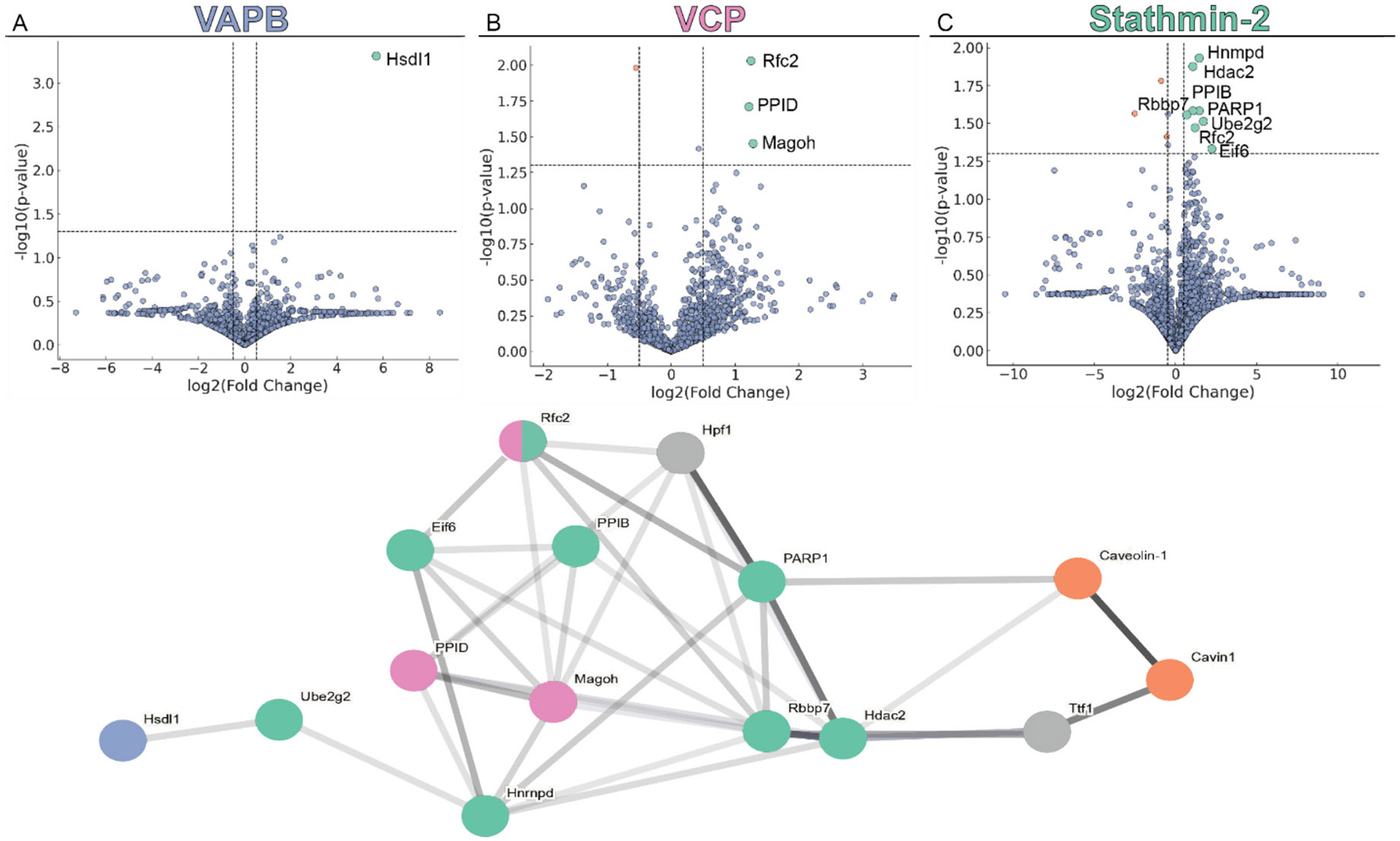
Differential gene expression analysis from the mass-spectrometry. Volcano plots display the distribution of differentially expressed genes in three conditions: (A) VAPB, blue circles, (B) VCP, pink circles, and (C) Stathmin-2, green circles. Genes with a ∣log_2_ fold change∣ > 0.5 and p-value <0.05 were considered significantly differentially expressed. Significantly upregulated genes are shown in green, downregulated in orange, and non-significant genes in blue. Other proteins that were not significant hits in the mass-spectrometry data are shown with grey or orange (Caveolin-1 and Cavin-1) circles. Line darkness corresponds to the confidence in the known connections according to the string database.

To determine how trimeric SOD1 drives ALS pathology through vesicle-based mechanisms, we stabilized trimeric SOD1 in cells (WT or FH SOD1 were overexpressed in NSC-34 cells) and performed co-immunoprecipitation (co-IP) mass spectrometry for VAPB, VCP, or Stathmin-2. This analysis revealed a distinct set of proteins that were entirely absent in WT SOD1-expressing controls, indicating that trimeric SOD1 actively alters vesicle formation, trafficking, and release. These proteins include Replication Factor C subunit 2 (Rfc2) (present in both the VCP and Stathmin-2 co-Ips), Protein mago nashi homolog (Magoh), Peptidyl-prolyl cis-trans isomerases B and D (PPIB, PPID), Poly [ADP-ribose] polymerase 1 (PARP1), Histone-binding protein (Rbbp7), Histone deacetylase 2 (Hdac2), Heterogeneous nuclear ribonucleoprotein D0 (Hrnpd), Eukaryotic translation initiation factor 6 (eIF6), Ubiquitin-conjugating enzyme E2 G2 (Ube2g2), and Inactive hydroxysteroid dehydrogenase-like protein 1(Hsdl1) (Fig. 5)(Supplementary Table S1). Many of these proteins were already known to directly or indirectly interact with VAPB, VCP, and Stathmin-2, all of which regulate ER integrity, axonal transport, and vesicle dynamics (McCluskey et al., 2022). For example, Hrnpd modulates Stathmin-2 mRNA stability, suggesting that trimeric SOD1 may interfere with axonal vesicle trafficking at the RNA level (Baralle and Romano, 2023; Bampton et al., 2020). Ube2g2, in collaboration with VCP, participates in ER-associated degradation and likely influences vesicle cargo turnover (Paubel et al., 2024; Araki and Kazuhiro, 2011). eIF6 plays a role in ER-Golgi transport and may regulate the flow of vesicle-bound proteins in response to trimeric SOD1 stress (Korneeva, 2022).

We also found that many of these hits converge on Caveolin-1/Cavin-1–regulated membrane domains, which govern a non-canonical vesicle trafficking pathway critical for endocytosis and microvesicle release (Busija et al., 2017). eIF6 may alter caveolar trafficking under trimeric SOD1-induced stress (Wang et al., 2022b; Anders et al., 2004). PPIB and PPID, which facilitate protein folding in the ER and Golgi (Blair et al., 2015; Koren et al., 2011), may help sort misfolded trimeric SOD1 into vesicle subtypes enriched in Caveolin-1. Ube2g2 likely targets damaged vesicle components for degradation (Koerver et al., 2019), a process coordinated by VCP at caveolar membranes (Ritz et al., 2011). Caveolin-1 has also been shown to modulate cellular stress responses through interactions with PARP1(Andrews and Rizzo, 2016), a nuclear enzyme activated by oxidative damage (Mao and Zhang, 2022). Under stress conditions, Caveolin-1 is required for the induction and cleavage of PARP1 in response to microparticle signaling (Andrews and Rizzo, 2016), highlighting a regulatory connection between caveolar domains and PARP1 activity. While PARP1’s primary role is in DNA repair (Mao and Zhang, 2022), its dependence on Caveolin-1 for activation suggests that caveolar compartments may help coordinate broader stress-adaptive responses, potentially influencing the trafficking or release of misfolded proteins such as trimeric SOD1. Rfc2, a DNA replication factor, may indirectly participate in stress signaling through its interaction with PARP1 and chromatin remodeling proteins, linking nuclear replication stress to cytoplasmic vesicle responses (Bütepage et al., 2018; Clarke and Mostoslavsky, 2022). Magoh, a component of the exon junction complex, regulates mRNA splicing and export (Mitra et al., 2023), potentially affecting vesicle-related transcripts such as Stathmin-2 and thereby altering downstream axonal vesicle trafficking. Rbbp7 and Hdac2 are part of nucleosome remodeling complexes that suppress gene expression, including stress-response genes; both have been linked to chromatin changes driven by oxidative stress (Zhan et al., 2024) and may interact functionally with PARP1 in modulating vesicle-associated genes. Hsdl1, though poorly characterized, may contribute to lipid metabolism (Zhang et al., 2011; Wingo et al., 2022) which is heavily involved in vesicle formation and regulated in part by Caveolin-1(Frank et al., 2008).

Although Histone PARylation Factor 1 (Hpf1) and Transcription Termination Factor 1 (Ttf1) were not detected in our proteomics data, STRING-db (Szklarczyk et al., 2025) network analysis revealed potential indirect associations between these proteins and both Caveolin-1–interacting partners and vesicle-regulating hits identified in this study. Hpf1 regulates the substrate specificity of PARP1(Kurgina et al., 2021), which is already implicated in caveolar stress signaling, suggesting a possible role in coordinating chromatin-initiated vesicle responses under oxidative stress. Ttf1, a chromatin-associated termination factor, has also been functionally linked to cellular stress responses and Cavin-1 function (Jansa, 1998; McMahon et al., 2019). Our findings support a model in which trimeric SOD1 hijacks Caveolin-1/Cavin-1–dependent vesicle machinery to enhance the release of extracellular vesicles enriched in toxic protein species. By altering vesicle content, release rates, and recycling dynamics, trimeric SOD1 likely promotes the spread of ALS pathology. These results highlight caveolar trafficking as a potential therapeutic target to alter the spread of ALS.

**Fig. 6.**
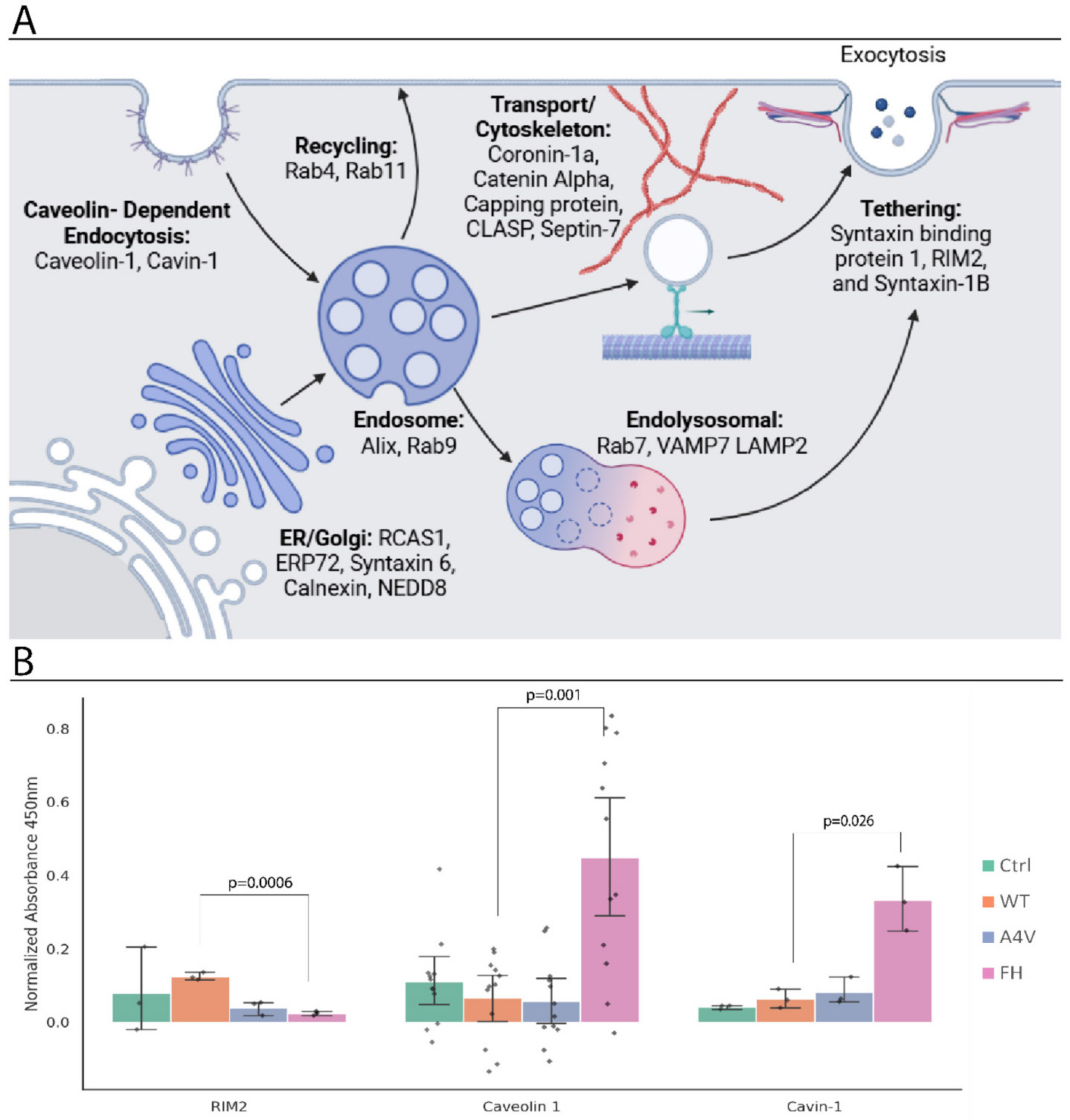
SOD1 trimer stabilization alters proteins involved in extracellular vesicle biogenesis and release. Proteins associated with distinct stages of EV biogenesis, membrane remodeling, and vesicle release were quantified on CD9+ EVs following incremental stabilization of trimeric SOD1. A) Twenty-two EV-related proteins were assessed using a sandwich ELISA to determine whether SOD1 trimer stabilization affects specific steps in EV formation or release. B) Only three proteins had significant changes with trimer stabilization and are presented as mean expression levels with individual replicates shown. RIM2 levels significantly decreased (p-value = 0.0006), while Caveolin-1 (p-value = 0.001) and Cavin-1 (p-value = 0.026) significantly increased with SOD1 trimer stabilization.

We also evaluated any changes to twenty-two EV-related proteins on EVs with trimer stabilization. These proteins can be partitioned into six main groups; ER and Golgi function (Viotti, 2016) (Calnexin (Bergeron et al., 1994), Endoplasmic Reticulum protein 72 kDa (ERP72)(Jansen et al., 2012), Receptor binding cancer antigen expressed on SiSo cells (RCAS1)(Xing et al., 2021), Syntaxin 6(Jung et al., 2012), and Neural precursor cell expressed, developmentally down-regulated 8 (NEDD8) (He et al., 2022)), lysosomal trafficking (Vesicle-associated membrane protein 7 (VAMP7)(Vats and Galli, 2022) and Lysosome-associated membrane protein 2 (LAMP2)(Ai et al., 2024; Qiao et al., 2023)), vesicle tethering (Syntaxin-1B, Rab-3 interacting molecule 2 (RIM2), and Syntaxin binding protein 1(Melland et al., 2021; Wang et al., 2022a)), Vesicle transport/ cytoskeleton (Coronin-1a (Rybakin and Clemen, 2005), Catenin-alpha (Kobielak and Fuchs, 2004), Capping protein (Burré and Volknandt, 2007), CLIP-associated protein (CLASP) (Lawrence et al., 2020), and Septin-7(Mostowy and Cossart, 2012)), proteins with direct involvement in endocytosis/ exocytosis (Alix (Larios et al., 2020), Caveolin-1, and Cavin-1(Busija et al., 2017; Chaudhary et al., 2014)), and Rab family proteins (Rab4, Rab7, Rab9, and Rab11) (Parakh et al., 2018). Together, these proteins coordinate the formation, cargo loading, and secretion of EVs (Fig. 6A). RIM2 (*p*-value = 0.0006 for the pariwise comparison of WT and FH) was the only protein tested with a significant decrease on EVs with trimer stabilization. RIM2 regulates Rab3, which directly controls vesicle fusion and exocytosis. The vesicle fusion and exocytosis are inhibited by both Manumycin A and GW4869(Wu et al., 2023), as well as vesicle transport on the cytoskeleton, which is connected to Stathmin-2(Krus et al., 2022). Although there are no direct roles yet known for RIM2 in ALS, RIM2 is crucial to multiple stages of EV release. Caveolin-1 (one-way ANOVA F = 12.24, *p* = 0.000006, pariwise comparison of WT and FH p-value = 0.001) and Cavin-1 (one-way ANOVA F = 22.94, *p* = 0.00028, pariwise comparison of WT and FH p-value = 0.026) had significant increases (Fig. 6B) on EVs with trimer stabilization. While some of the other proteins tested were significant using one-way ANOVA, none of the other proteins had significant incremental pairwise changes (Supplementary Fig. S5).

**Fig. 7.**
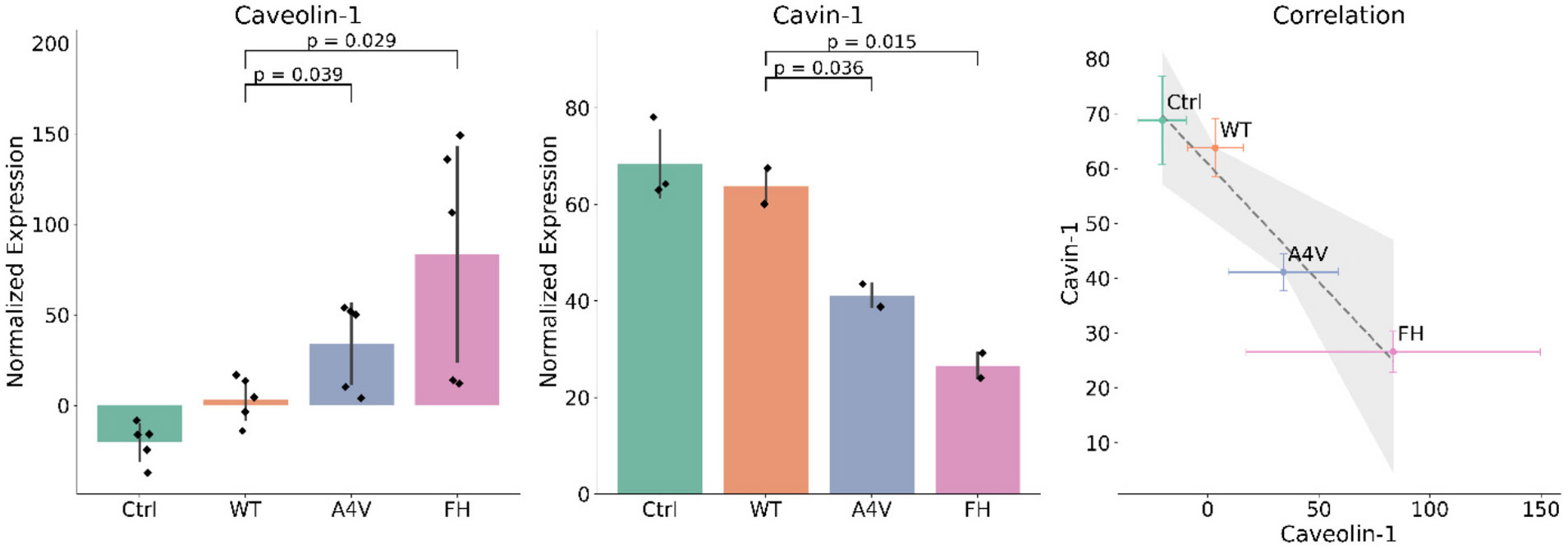
Native western blotting for Caveolin-1 and Cavin-1 displays alternate effects in cell lysate. Caveolin-1 oligomers increase with trimer stabilization (WT to A4V p-value = 0.039, WT to FH p-value =0.029), while Cavin-1 oligomers decrease with trimer stabilization (WT to A4V p-value = 0.036, WT to FH p-value =0.015). Caveolin-1 and Cavin-1 levels show a strong inverse correlation (Pearson's correlation coefficient *r* = −0.98, *p* = 0.021).

Cavin-1 oligomers decrease in cell lysate with trimer stabilization, and oligomers of Caveolin-1 increase with trimer stabilization, while the overall levels of Cavin-1 and Caveolin-1 were not affected (Fig. 7, full blots in Supplementary Fig. S8 prominent bands were quantified from triplicate blots). This shift in oligomerization reveals a strong inverse correlation between Caveolin-1 and Cavin-1 oligomers (Pearson *r* = −0.98), suggesting that trimeric SOD1 disrupts the formation of functional Caveolin-Cavin complexes. These Caveolin-1 oligomers are obvious in native western blots, but break down rapidly with the addition of 0.1 M Dithiothreitol (DTT) and heat (Supplementary Fig. S6). Since both Caveolin-1 and Cavin-1 require oligomerization to form mature caveolae (Busija et al., 2017), this imbalance suggests that Caveolin-1 may be forming aberrant oligomers incapable of recruiting Cavin-1. Caveolin-1 and Cavin-1 are both inhibited by Genistein and directly interact to regulate Caveolin-dependent endocytosis. This may reflect a loss of proper membrane microdomain organization, where trimeric SOD1 alters the lipid or trafficking environment needed for stable Cavin-1 association. Caveolin-1 specifically has been recently connected to ALS (Cooper-Knock et al., 2020); overexpression of Caveolin-1 in an ALS SOD1 mouse model improved neuromuscular function (Sawada et al., 2019), suggesting a loss of function of Caveolin-1 may be contributing to ALS disease pathology (Benarroch, 2025), which could be occurring due to trimeric SOD1 oligomerizing with Caveolin-1. Additionally, Ritz et al. previously showed that VCP directly affects ubiquitylation of Caveolin-1, leading to altered Caveolin-1 oligomers similar to what we observed with trimer stabilization (Ritz et al., 2011). No other proteins that are altered by trimeric SOD1 stabilization had significant expression changes in NSC-34 cell lysate (For Spg11, the FH expression was not significantly increased compared to WT; we are unsure why the A4V expression was low, Supplementary Fig. S7) (Supplementary Fig. S8). The combined evidence for the role of the Caveolin-1 pathway, as well as the RIM2-mediated exocytosis pathway in the release of altered EVs with trimeric SOD1 stabilization, suggests multiple pathways are engaged by contributing to a joint undiscovered novel pathway for EV release and spreading in ALS.

## 3. Methods

### 3.1. Lysate and EV isolation

We cultured neuroblastoma spinal cord hybrid cells (NSC-34) in six-well plates to 60–80% confluency using 50:50 DMEM:F12 medium supplemented with 10% (*v*/v) fetal bovine serum and 1% penicillin/streptomycin. We differentiated the NSC-34 cells into a neuronal phenotype by adding retinoic acid to a final concentration of 10 μM (Hnath and Dokholyan, 2022) for 48 h. Once the cells were differentiated, we transiently transfected WT SOD1 or mutants (A4V or FH (Hnath and Dokholyan, 2022)) using lipofectamine (JetOptimus) in triplicate wells for each mutant (and triplicate control wells that were treated with the transfection reagent and no DNA). Twenty-four hours after transfecting, we switched the medium and allowed the cells to grow for an additional 48 h, at which point we collected the enriched medium and lysed the cells using RIPA buffer with protease inhibitors. We performed western blotting using the lysed cell samples to confirm transfection of the different ALS protein mutants and to evaluate any changes in other protein levels within the cells due to SOD1 overexpression.

Immediately after collecting the cell medium, we centrifuged the enriched medium at 1000 rpm to pellet any floating cells. We then transferred the supernatant to an ultracentrifuge tube and centrifuged for two hours at 100,000 x g to pellet the EVs (Théry et al., 2018b; Witwer et al., 2021; Gomes et al., 2007). We resuspended the EV pellet in 1× PBS (50 μL PBS per well of medium) and used it immediately for either ELISA or western blotting. We added RIPA buffer at a 1:1 ratio to any EV pellet samples before western blotting.

### 3.2. Western blotting

All samples were normalized before western blotting by calculating the total protein concentration using a Bicinchoninic acid assay (BCA). All western blots were performed using 12% protein gels cast using the Mini-PROTEAN Tetra Handcast System (BioRad) and performed using a Mini-PROTEAN Tetra Vertical Electrophoresis Cell (BioRad) in Trisglycine running buffer (with or without SDS, depending on native or reduced). Reduced samples were prepared using SDS-PAGE loading dye with 0.1 M DTT. We performed all blotting onto PVDF membranes using the BioRad Transblot turbo system before being blocked in 5% BSA solution (diluted in 1× TBST). All blots were incubated in primary antibody overnight at 4 °C and in secondary antibody for 1 h at room temperature before development using SuperSignal West Pico PLUS Chemiluminescent substrate and imaging using a Chemidoc imager (BioRad). The western blot images were quantified using ImageJ, and unpaired *t*-tests were performed using Python. Spg11 bands ran lower than the expected molecular weight when lysed (around 180 kDa, opposed to the expected 280 kDa), most likely due to the gel percentage used being more optimal for lower molecular weight proteins. Cavin-1 consistently appeared as a dimer on reduced gels across multiple replicates. Rim2 only appeared as a small fragment on reduced gels, which could correspond to part of the Zn-finger domain (11 kDa) or the PDZ domain (12 kDa), whole RIM2 should be 180–200 kDa, which is seen in the native blot.

**Table:** 

Antibody	Company	Catalog Number
ALIX	Cell Signaling	2171
ALS2	Proteintech	13,998–1-AP
BiP	Cell Signaling	3177
C4F6	Medimabs	MM-0070-2-P
C9orf72	Proteintech	22,637–1-AP
Capping protein (Rb anti CARMIL2)	My Bio Source	
CAV1	Cell Signaling	3267 T
Cavin-1	Cell Signaling	69,036 T
CD9	VWR	10,084–398
CDK5	Proteintech	10,430–1-AP
CHMP2B	Proteintech	12,527–1-AP
CLASP2	Cell Signaling	14629S
Coronin-1a	Cell Signaling	92,904 T
ER Golgi Sampler Kit	Cell Signaling	12,718
Fig. 4	Fisher	NB095247
FUS/TLS	Proteintech	11,570–1-AP
GS28	VWR	10,087–726
LAMP2	My Bio Source	MBS9607375
NEDD8	Proteintech	16,777–1-AP
NSFL1 cofactor p47	Fisher	PIPA521633
OPTN	Proteintech	10,837–1-AP
Rab Family Antibody Sampler kit	Cell Signaling	9385 T
RIM2	Proteintech	20,093–1-AP
Septin 7	Abcam	ab186021
sequestosome-1	Cell Signaling	5114
SOD1	Proteintech	67,480–1-Ig
SPG11	Proteintech	16,555–1-AP
Stathmin-2	Cell Signaling	56,016
Syntaxin 1A/B	VWR	66,437–1-IG
Syntaxin binding protein 1	Fisher	89,018,297
TBK1	Cell Signaling	3504 T
TDP-43	Proteintech	10,782–1-AP
UBQLN2	Proteintech	23,449–1-AP
VAMP7	Fisher	PIPA5116892
VAPB	VWR	76,464–334
VCP	Cell Signaling	2648
Anti-Rabbit HRP	Fisher	A16096
Anti-Rat HRP	Fisher	AP202PMI
Anti-Mouse HRP	Fisher	AP124PMI

### 3.3. ELISA

We performed sandwich ELISAs using anti-CD9 as the capture antibody (Witwer et al., 2021; Witwer et al., 2021) to isolate EVs, then anti-C4F6 (detects trimeric SOD1(Proctor et al., 2016)) or antibodies against other ALS-related proteins FUS, TDP-43, C9orf72, VAPB, SQSTM1, UBQLN2, VCP, OPTN, TBK1, ALS2, CHMP2B, SPG11, Stathmin-2, NEK1, FIG. 4, NSFL1, and Cdk5 (or other EV-related proteins) as the detection antibody. Rat anti-CD9 was coated at 1 mg/mL and 50 μL/well (diluted in 1× PBS) overnight at 4C. Following coating we washed the plates 3 times with 1× PBS, then blocked the plate using 1% BSA overnight at 4 °C. The following day, we added isolated EVs (50 μL/ well) to the wells and incubated them for 2.5 h at 37 °C. Then we washed the plates 3 times with 1× PBS and added primary antibodies (1:1000 for all except C4F6 which was used at 1:250) before leaving the plates at 4 °C overnight. Next, we washed the plates 3 times with 1× PBS and added 50 μL of secondary antibody (anti-Rabbit HRP or anti-Mouse HRP) to each well at 1:1000 for 1 h at room temperature. After a final wash, we visualized the HRP signal by adding 100 μL TMB substrate (per well) for 5 min, then stopped the reaction by adding 50 μL of 2 N Sulfuric acid. We then quantified the absorbance at 450 nm for each well using a SpectraMax i3 plate reader.

To account for different concentrations of EVs, we performed an indirect ELISA alongside the sandwich ELISA to quantify the amount of CD9+ vesicles in each sample. We coated ELISA plates with isolated EV samples (diluted 1:5 in 1× PBS) overnight at 4 °C. The following morning, we washed the plates 3 times with 1× PBS, then added Rat anti-CD9 antibody (1:1000) for 1 h at room temperature. We then washed the plates again and added an anti-Rat HRP secondary antibody for 1 h at room temperature. After a final washing step, we used TMB substrate to quantify the HRP signal as described previously.

### 3.4. Analysis and statistics

We analyzed the final ELISA signal by first subtracting the negative control (same process for sandwich ELISA but no EVs added) signal from the sandwich ELISA signal, then dividing by the CD9 signal to normalize each well to the total concentration of EV-positive EVs. The control samples are from NSC-34 cells with a mock transfection (transfection reagent was added without DNA). All ELISA sample intensities were normalized to both the signal intensity of triplicate negative control wells (all antibodies added with no sample) as well as to the total concentration of CD9 protein within each sample to normalize for total EV concentration; thus, the sample intensity is negative in some cases. The overexpression of SOD1 between the different mutants was confirmed to be consistent using western blotting for total human SOD1 (Supplementary fig. S1), and none of the significantly changed proteins had any changes in expression within cells with trimer stabilization (Fig. 7), the only changes were on EVs. We performed a one-way ANOVA test for the four overexpression conditions of each protein (control, WT, A4V, FH) then calculated the *p*-values for any differences between groups using a Tukey honest significant difference test for any proteins with a significant ANOVA using Python. A protein was categorized as a “hit” if there was a significant increase or decrease between WT and A4V or WT and FH following a trend of increase or decrease across the groups.

### 3.5. EV mechanism inhibitors

We grew NSC-34 cells and transfected them with SOD1 mutant constructs following the methods described above. Post-transfection we changed the medium and add DMSO (2 μL/well, vehicle control), Manumycin A (1 μM, ESCRT-dependent exocytosis inhibitor, VWR 102513–528), GW4869 (35 μM, ESCRT-independent exocytosis inhibitor, VWR 103548–206), Wortmannin (25 nM, macropinocytosis inhibitor, VWR 102515–748), EIPA (50 μM, Na/H channel inhibitor, also a factor in macropinocytosis, Sigma A3085–25 mg), Chlorpromazine (25 μM, Clathrin-dependent endocytosis inhibitor, VWR 80055–146), Genistein (75 μM, Caveolin-dependent endocytosis inhibitor, VWR 89148–900), or Mitmab (10 μM, Dynamin Inhibitor, Fisher 32–4411500MG) for 48 h, then we isolated EVs and performed an ELISA as described above. We determined all inhibitor concentrations by testing five different concentrations within the recommended range of each inhibitor and choosing the highest concentration that did not cause cell death (determined by visualizing the cells on a Keyence BZ-X810 inverted microscope). Alongside the imaging, we also performed a lysed EV western blot for the Manumycin A and GW4869 concentration curves for both CD9 and CD63; we chose concentrations that lowered the level of CD63 but did not affect CD9. The fold change was calculated for each inhibitor (compared to the vehicle control), and the error bars were determined by calculating the absolute error using Microsoft Excel.

### 3.6. Co-IP mass spectrometry proteomics

We grew NSC-34 cells transfected with either WT or FH SOD1 (following the methods described above). We lysed the cells (RIPA buffer with protease inhibitors, triplicate wells) and combined samples of the lysate with antibodies for either VAPB, VCP, or Stathmin-2. After incubating at 4 °C overnight, we added Protein G Magnetic Beads and allowed the mixture to incubate at room temperature for two hours. We then separated the beads from the supernatant using a magnet and washed the beads three times with mass spectrometry grade 1× PBS (Supplementary Fig. S3). Finally, all liquid was removed from the beads, and they were frozen at −80 °C before shipping them to the University of Michigan for on-bead trypsin digestion, 90-min shotgun Mass Spectrometry proteomics using an OrbiTrap Ascend Tribrid with ETD and FAIMS, and analysis using Proteome Discoverer v3.0. Control samples, including cell lysate and beads without any antibody, are also analyzed to eliminate any non-specific binding to the magnetic beads (Supplementary Fig. S4).

Following mass spectrometry analysis, the datasets for control, Stathmin-2, VAPB, and VCP samples were cleaned by removing empty values. Subsequently, a statistical *t*-test was employed to compare mutant samples against the control, generating p-values for each protein. Volcano plots were then constructed to visualize these results, enabling the identification of significantly upregulated (Supplementary Table S1) and downregulated (Supplementary Table S2) proteins based on their log2 fold change. Further, we identified any protein pathways that were altered between the WT and FH samples in all three antibodies using the STRING database (Szklarczyk et al., 2025). All mass spectrometry data was uploaded to Zenodo, doi:https://doi.org/10.5281/zenodo.15650245.

## 4. Discussion

ALS is currently diagnosed by ruling out other conditions (Goutman et al., 2022), which makes the process long and challenging. This delay has severe implications for patient outcomes. Without a definitive test, patients often spend months or even years undergoing evaluations before receiving a diagnosis. By that time, the disease has typically progressed, limiting treatment options and reducing the potential benefit of therapeutic interventions. Delayed diagnosis contributes to the reduced life expectancy seen in ALS, as patients are often diagnosed too late for meaningful disease-modifying treatments to take effect (Abrahams, 2023; Falcão De Campos et al., 2023; Goutman et al., 2022). Identifying shared factors involved in ALS development could improve our understanding of the disease, guide therapy development, and lead to better diagnostic tools. Proteins found on extracellular components like EVs are promising as clinical biomarkers because they can be measured in biofluids rather than requiring invasive tissue samples (Darabi et al., 2024). Finding a common pathway responsible for the release of altered EVs in ALS could also reveal new targets to slow or stop disease progression. Many studies have shown that EVs play a role in how ALS spreads in the body (Afonso et al., 2023; Anakor et al., 2021), suggesting that they are altered in a way that allows the disease to move between cells. Previous research has mostly focused on how common ALS-related mutations affect the spread of misfolded SOD1 on EVs (Grad et al., 2014). Other studies have shown that proteins like FUS and TDP-43 (both commonly mutated in ALS) can promote the appearance of misfolded SOD1 on EVs (Pokrishevsky et al., 2016). This work takes a new approach by showing that misfolded (trimeric) SOD1 on EVs can, in turn, alter the levels of several ALS-associated proteins on EVs. These findings suggest a network of ALS proteins linked through EV-related disease spread, which were previously thought to act independently.

The field of EV research (especially small EVs like exosomes) has only gained traction within the last decade and there is still very little known about EV formation and release (Couch et al., 2021), especially in connection to disease states. Studying EVs can be difficult as methods are still evolving, despite using the same conditions on multiple repeats of the assays included in this work there was still a large amount of variability potentially due to; differences in the passage number of each cell group used, time between ultracentrifugation and isolating the pellet (vesicle pellets typically stayed within the tube when removing the media but would occasionally begin dispersing back into the solution with any slight movement), and vesicle concentrations for the ELISA assays (although we scaled the cell plating to the amount of antibodies being tested for each ELISA, scaling experiments can cause unforeseen changes in the pelleting of vesicles). We accounted for these variables by normalizing all ELISAs to the total concentration of CD9+ vesicles within each sample. We chose CD9 to isolate vesicles since numerous previous studies have shown the colocalization of CD9 with misfolded SOD1 on EVs (Silverman et al., 2019; Gomes et al., 2007), using a sandwich ELISA approach with a CD9 capture prevents us from losing large amounts of sample while attempting to purify vesicles. Additionally, following the minimal information for the study of EVs (MISEV) criteria the isolated EVs were positive for tetraspanins (CD9, CD81, and CD63) as well as other cytosolic proteins (Alix, Caveolin-1, and ChMBP2b) to confirm the isolated samples were enriched in EVs (Witwer et al., 2021; Théry et al., 2018a).

**Fig. 8.**
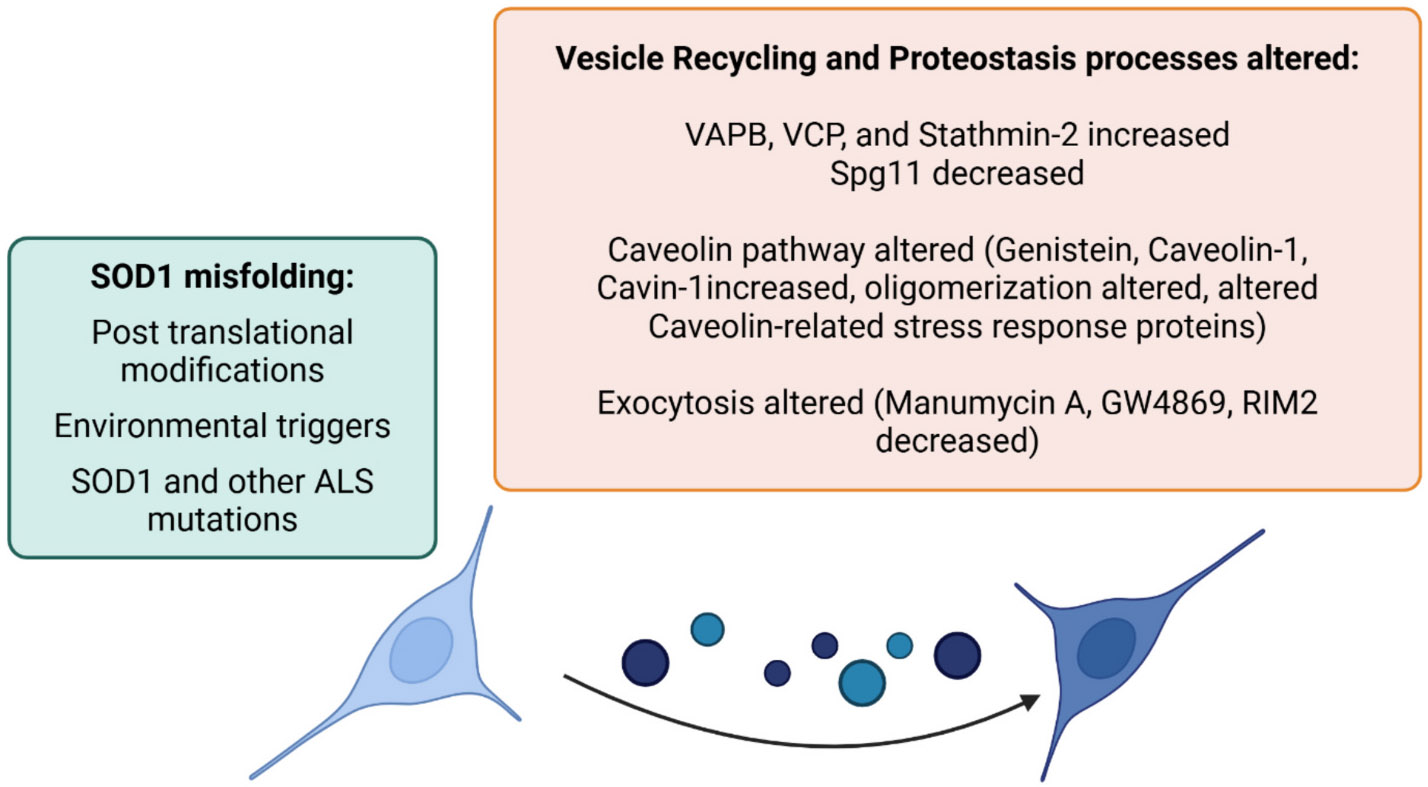
Stabilization of trimeric SOD1 alters EVs by potentially affecting vesicle recycling and proteostasis.

Stabilization of trimeric SOD1 altered the levels of VAPB, VCP, Stathmin-2, SPG11, RIM2, Caveolin-1, and Cavin-1 on EVs—proteins that are all interconnected at various stages of EV biogenesis and release (McCluskey et al., 2022; Busija et al., 2017; Wu et al., 2023). Although the mechanistic details of EV biogenesis remain incomplete, our data suggest these proteins may converge within a common vesicle trafficking and proteostasis pathway that is dysregulated in ALS (Fig. 8). Supporting this model, we found that VAPB, VCP, and Stathmin-2 levels significantly increased on EVs in the presence of trimeric SOD1, while SPG11 levels decreased, indicating a selective reprogramming of vesicle content. The upregulation of VAPB and VCP, both essential regulators of ER and endolysosomal trafficking (Ritz et al., 2011; Miranda and Di Paolo, 2018), suggests that trimeric SOD1 directs these proteins into EV-associated pathways, enhancing the release of proteostasis-related cargo. Macropinocytosis inhibitors (wortmannin and EIPA) further amplified EV levels of VAPB, VCP, and Stathmin-2, implying that when compensatory uptake mechanisms are blocked, SOD1-driven EV secretion is intensified, possibly as a cellular stress response. In contrast, genistein, a Caveolin-1 inhibitor, suppressed all three proteins on EVs, implicating Caveolin-dependent trafficking in this process. Interestingly, Manumycin A and GW4869, which inhibit ESCRT-independent EV release, did not affect VAPB or VCP levels but selectively reduced Stathmin-2, suggesting that distinct EV biogenesis routes may be exploited differently by trimeric SOD1.

To further explore the mechanistic drivers of this SOD1 trimer-induced vesicle alterations, we performed co-immunoprecipitation mass spectrometry for VAPB, VCP, or Stathmin-2 from cells expressing stabilized trimeric SOD1. This analysis revealed a set of proteins absent in WT SOD1-expressing controls but selectively enriched in the trimer condition, including RFC2, Protein mago nashi homolog, PARP1, RBBP7, HDAC2, hnRNPD, eIF6, UBE2G2, and Inactive hydroxysteroid dehydrogenase-like protein 1. Many of these proteins regulate ER-Golgi vesicle trafficking, protein folding, or membrane remodeling, and several localize to Caveolin-rich domains. In particular, eIF6 and PPID are linked to ER-to-Golgi transport (Wang et al., 2022b; Anders et al., 2004; Blair et al., 2015; Koren et al., 2011), while UBE2G2 and PARP1 interact with VCP (Araki and Kazuhiro, 2011) and stress-induced vesicle release (Andrews and Rizzo, 2016; Mao and Zhang, 2022). Genistein also inhibits HDAC2(Sundaram et al., 2018) and may therefore disrupt the expression of essential factors that support Cavin-1 oligomerization or membrane domain integrity, potentially compounding the destabilizing effects of trimeric SOD1 on caveolar vesicle machinery. Their enrichment supports a model where trimeric SOD1 repurposes Caveolin-1/Cavin-1–dependent pathways to drive altered EV production and export.

Caveolin-1 and Cavin-1 levels also increased on EVs with trimer stabilization, while RIM2, a regulator of synaptic vesicle exocytosis (Wu et al., 2023), decreased, indicating a shift in vesicle origin or release mechanism. Within cells, we observed a marked increase in oligomeric Caveolin-1 and a corresponding decrease in Cavin-1 oligomers, consistent with the formation of abnormal Caveolin-rich membrane domains and displacement of Cavin-1. Because Cavin-1 requires oligomerization to support caveolae formation and vesicle curvature, its displacement suggests a loss of functional membrane scaffolding (Busija et al., 2017). This imbalance may result in membrane deformation, defective vesicle sorting, and altered fusion dynamics. Together, these findings suggest that trimeric SOD1 not only disrupts intracellular proteostasis but also hijacks membrane trafficking machinery, altering EV composition and promoting the spread of pathology. The accumulation of trimeric SOD1 aligns with elevated oligomeric Caveolin-1 within cells, supporting a model in which misfolded SOD1 reshapes membrane domains and reroutes vesicle export pathways. Given the known roles of VAPB, VCP, Caveolin-1, and Cavin-1 in virus budding and vesicle system hijacking (Xing et al., 2020; Dorsch et al., 2021; Waisner and Kalamvoki, 2019; Das and Dudley, 2021), a toxic gain-of-function mechanism is likely at play. This viral-like exploitation of the endocytic and exocytic machinery may explain the broad convergence of disrupted pathways in ALS. The proteomic alterations described above expand this model by identifying specific vesicle- and proteostasis-related proteins uniquely altered in the presence of trimeric SOD1. Our results support the existence of a unified trafficking axis, driven by toxic protein assemblies, that facilitates disease spread and offers novel targets for therapeutic and biomarker development (Fig. 8).

## Supplementary Material

supporting information

## Data Availability

Data will be made available on request.

## Appendix A. Supplementary data

Supplementary data to this article can be found online at https://doi.org/10.1016/j.nbd.2026.107309.
