# Supplementary material for: Novel extracellular vesicle release pathway facilitated by toxic superoxide dismutase 1 oligomers
Source: Neurobiol Dis. Author manuscript; Available in PMC 2026 Apr 9. (PMC13064919; doi:10.1016/j.nbd.2026.107309)
Supplement: supporting information [file NIHMS2158866-supplement-supporting_information.pdf]

Supplementary Information: Novel extracellular vesicle release pathway facilitated by toxic superoxide dismutase 1 oligomers

Brianna Hnath[1,2], Srinivasan Ekambaram[1], and Nikolay V. Dokholyan[1,2,3]

[1]Department of Pharmacology, Penn State College of Medicine, Hershey, PA, USA
[2]Department of Biomedical Engineering, Pennsylvania State University, University Park, PA, USA
[3]Department of Neurology, The University of Virginia, Charlottesville, VA, USA

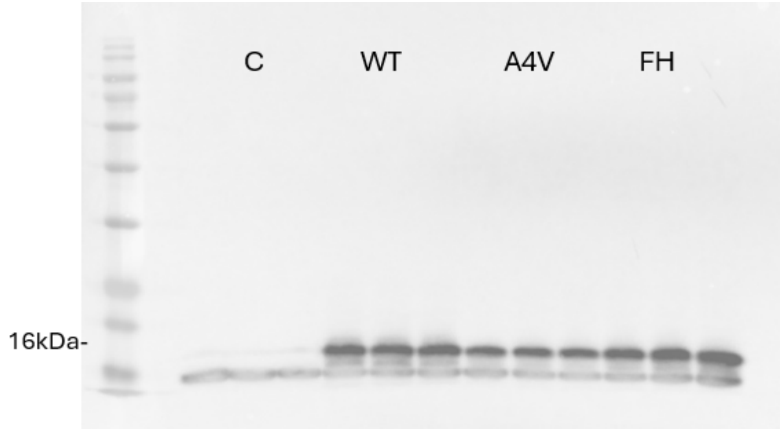

Figure S1. Western blots of NSC-34 lysate overexpressing SOD1 WT or mutants (A4V or FH).

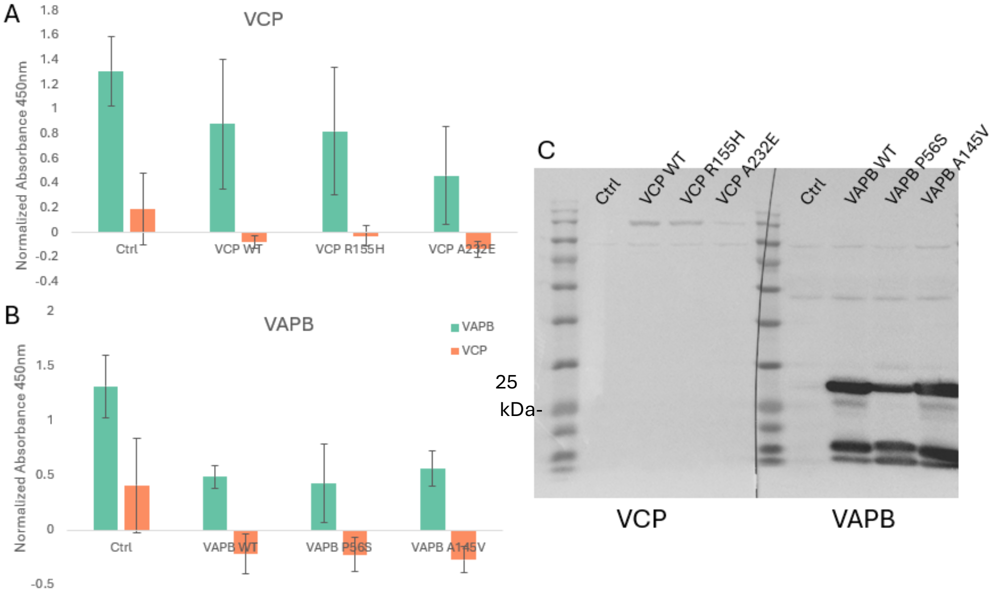

Figure S2. Overexpression of VAPB, VCP mutants does not induce the same increases in VAPB and VCP on CD9+ vesicles that is observed with trimeric SOD1 stabilization. Overexpression of the VCP and VAPB mutants in NSC-34 cells was confirmed using western blotting.

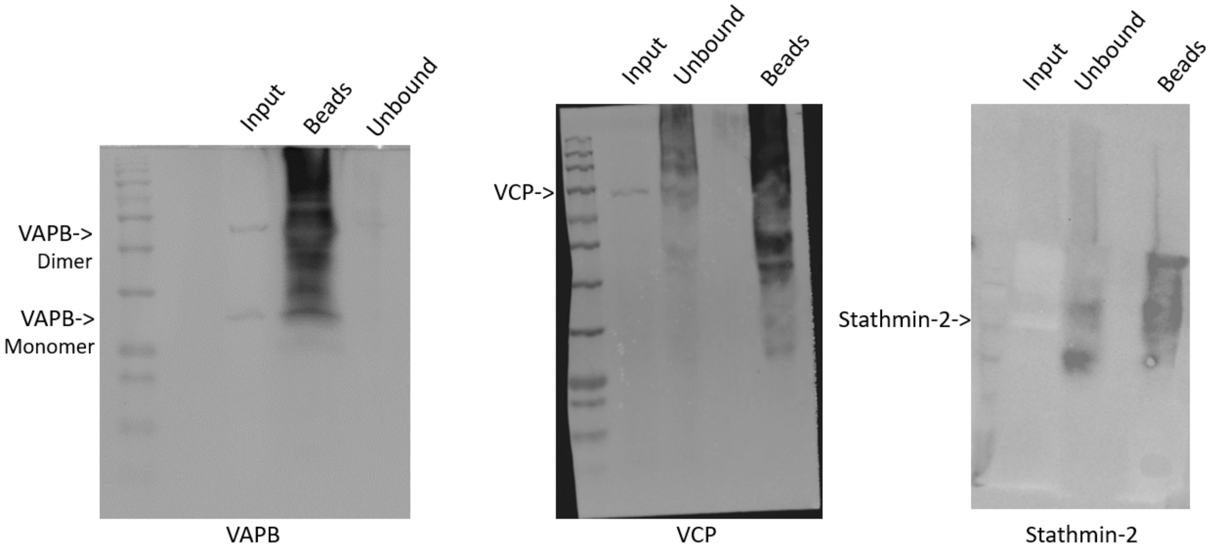

Figure S3. Western blotting of the input cell lysate, magnetic beads, and unbound fraction from the pulldowns confirms that the expected proteins were bound to the beads. Strong higher bands on the bead fractions are from the antibody.

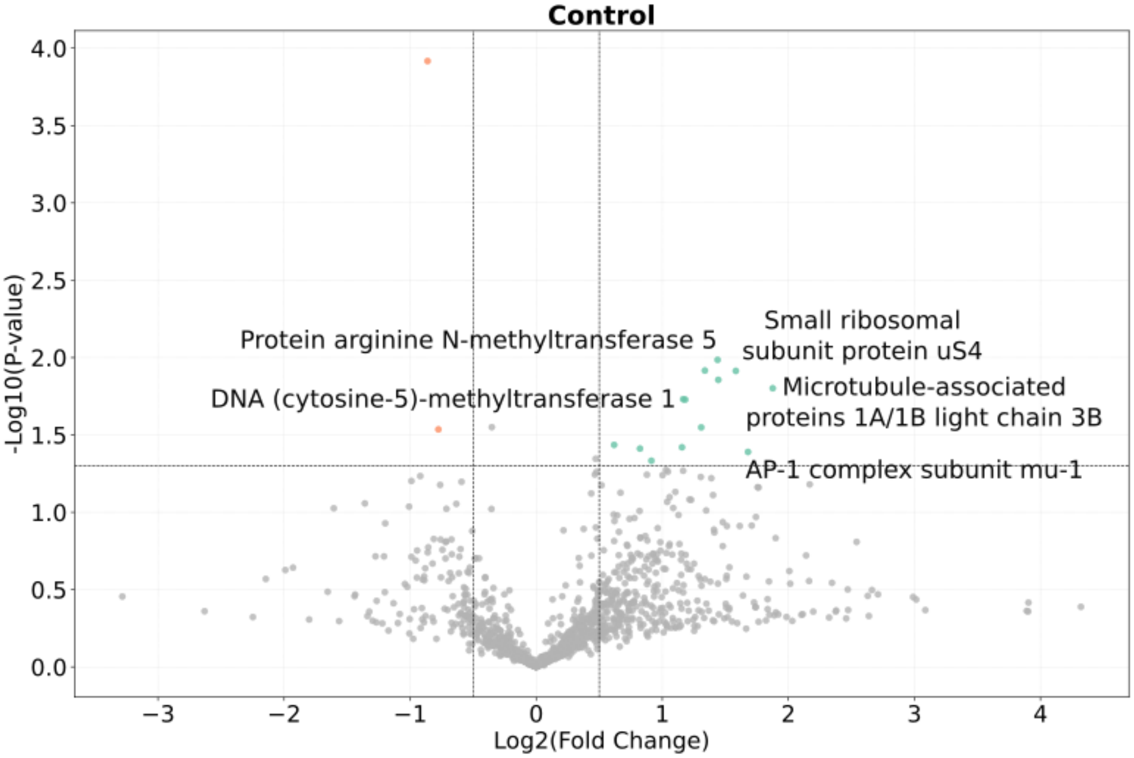

Figure S4. Differential gene expression analysis from the mass-spectrometry negative controls. Volcano plot displays the distribution of differentially expressed genes in the bead control. Genes with a $|\log_2$ fold change$| > 0.5$ and p-value < 0.05 were considered significantly differentially expressed. Significantly upregulated genes are shown in green, downregulated in orange, and non-significant genes in grey. These results were eliminated from the antibody Co-IP results.

Control	VCP	Stathmin-2	VAPB
Eukaryotic translation initiation factor 2 subunit 2	Replication factor C subunit 2	Poly [ADP-ribose] polymerase 1	Inactive hydroxysteroid dehydrogenase-like protein 1
Small ribosomal subunit protein uS3	Protein mago nashi homolog	Peptidyl-prolyl cis-trans isomerase B	
Small ribosomal subunit protein uS4	Peptidyl-prolyl cis-trans isomerase D	Histone-binding protein RBBP7	
Small ribosomal subunit protein uS9		Histone deacetylase 2	
Proliferation-associated protein 2G4		Replication factor C subunit 2	
WD repeat-containing protein 48		Heterogeneous nuclear ribonucleoprotein D0	
Large ribosomal subunit protein eL34		Eukaryotic translation initiation factor 6	
AP-1 complex subunit mu-1		Ubiquitin-conjugating enzyme E2 G2	
Microtubule-associated proteins 1A/1B light chain 3B			
DNA (cytosine-5)-methyltransferase 1			
Chitinase domain-containing protein 1			
Protein arginine N-methyltransferase 5			
Ras-related protein Rab-23			

Table S1. Proteins that were significantly upregulated with trimer stabilization.

Control	VCP	Stathmin-2
Tropomyosin alpha-4 chain	F-actin-capping protein subunit alpha-1 [OS=Mus musculus]	Lysine-specific demethylase 9 [OS=Mus musculus]
Transcription initiation factor TFIID subunit 10		Pyrroline-5-carboxylate reductase 1, mitochondrial [OS=Mus musculus]
		Microtubule-associated serine/threonine-protein kinase 3 [OS=Mus musculus]

Table S2. Proteins that were significantly downregulated with trimer stabilization. VAPB had no significantly downregulated proteins.

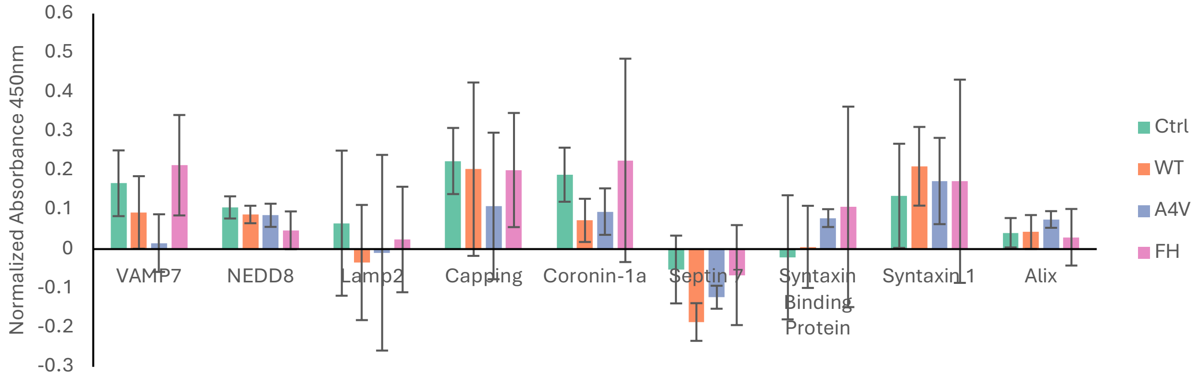

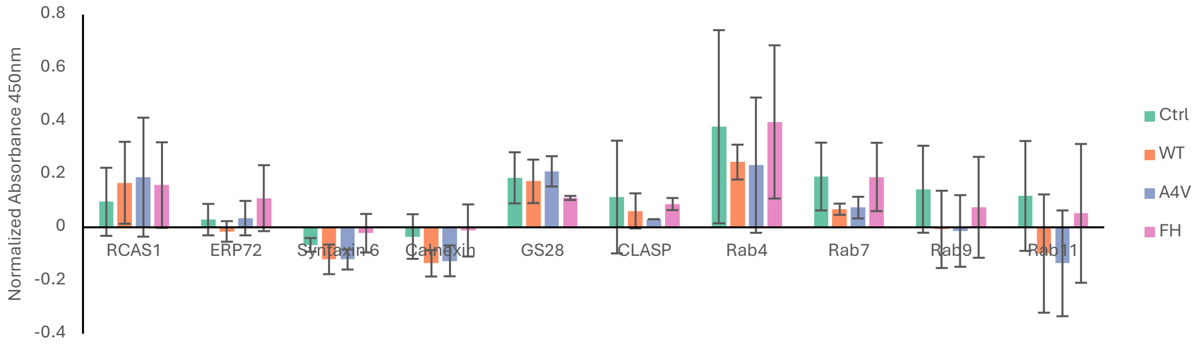

Figure S5. Alterations on twenty-two EV-associated proteins were tested on EVs with the stabilization of trimeric SOD1, these nineteen proteins had no significant changes.

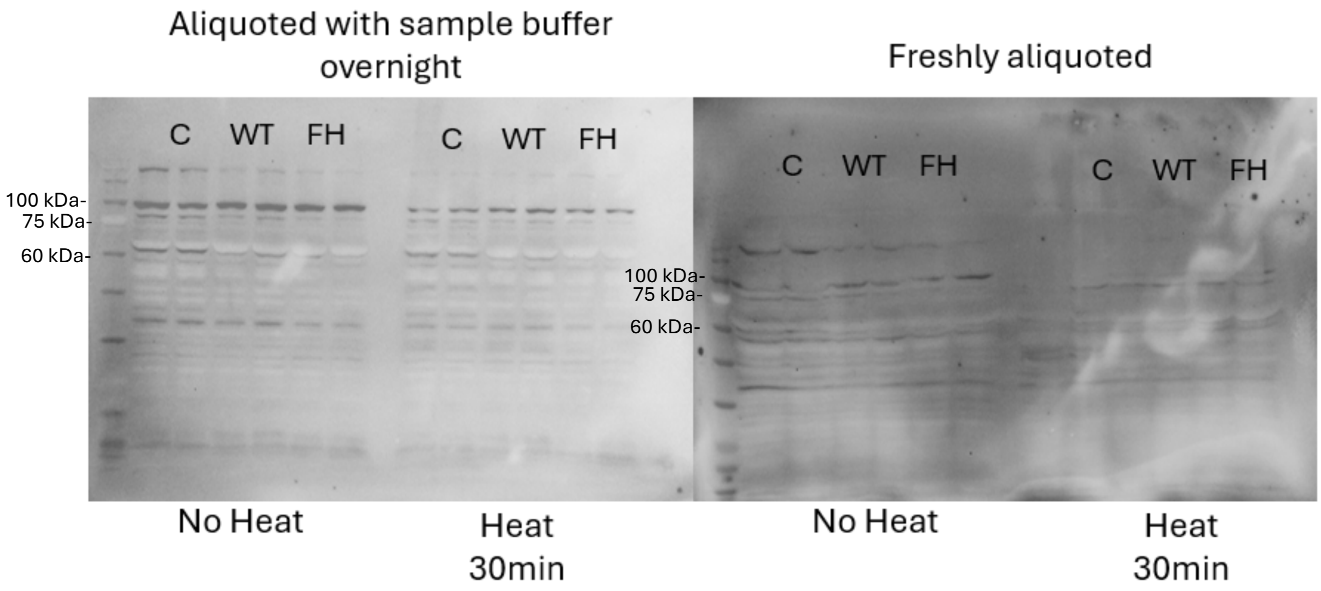

Figure S6. Stabilization of trimeric SOD1 (FH) promotes increases in oligomeric Caveolin-1 species that are also observed in native blots. Oligomeric Caveolin-1 breaks down or forms different oligomers after overnight incubation in sample buffer (0.1M DTT) and after heating the samples.

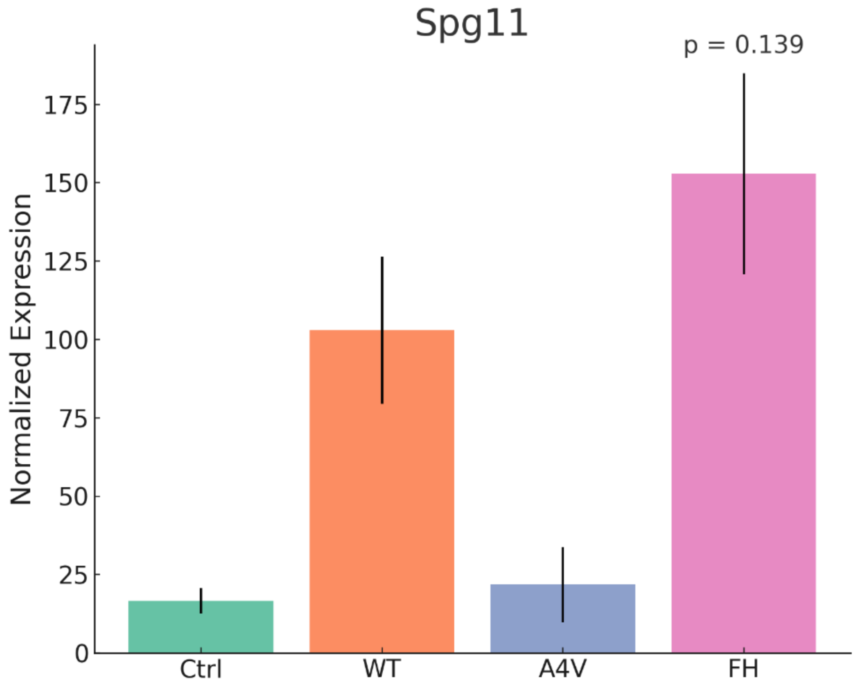

Figure S7. Spatacsin (Spg11) native western blot quantification did not have a significant increase in oligomers between WT and FH SOD1.

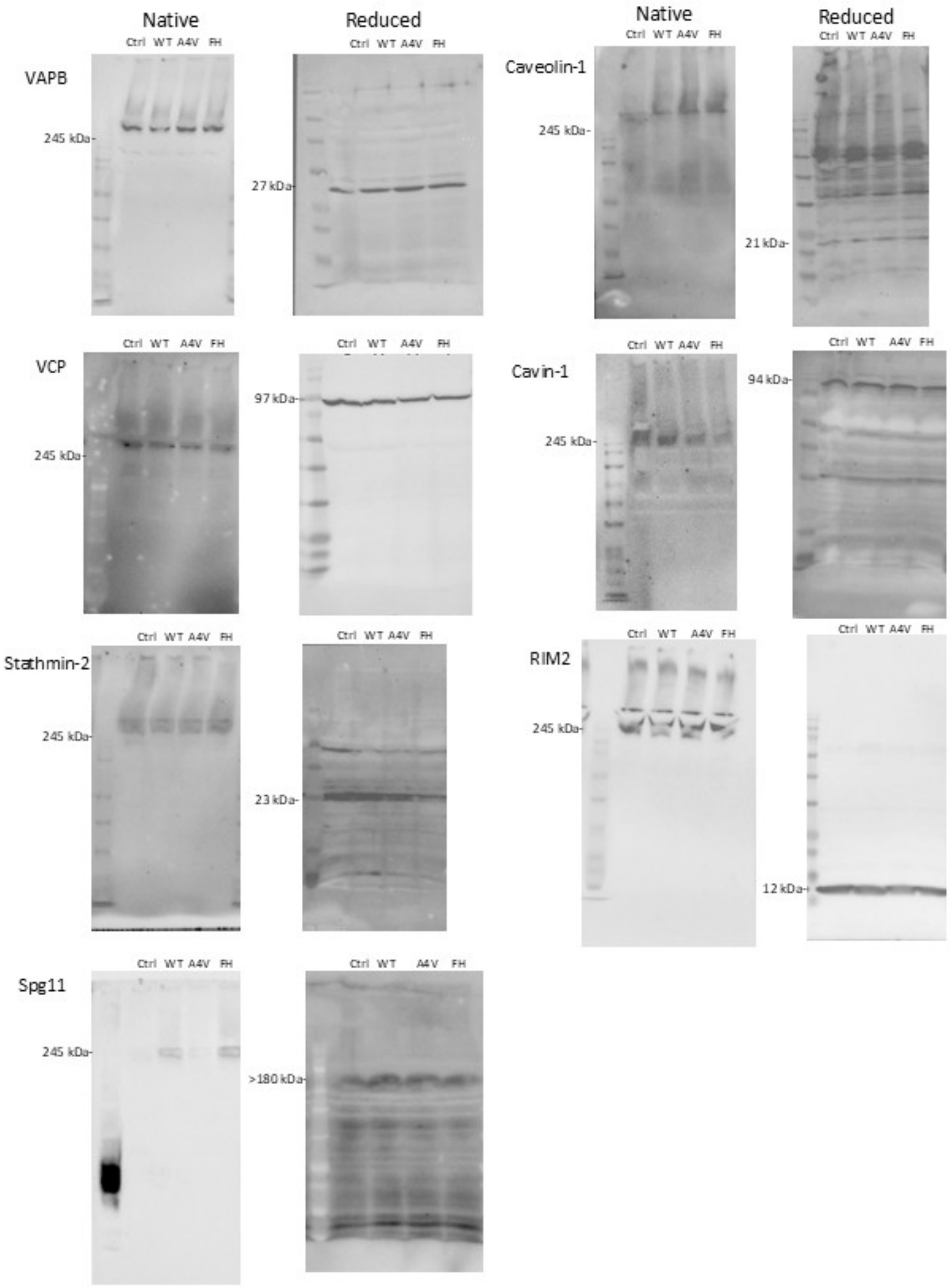

Figure S8. Full native and reduced Western blots of cell lysate from NSC-34 cells overexpressing WT, A4V, or FH SOD1.
